# Promoting Alzheimer’s disease research and therapy with stem cell technology

**DOI:** 10.1186/s13287-024-03737-w

**Published:** 2024-05-07

**Authors:** Zimeng Cao, Fanshu Kong, Jiaqi Ding, Chunxia Chen, Fumei He, Wenbin Deng

**Affiliations:** 1 School of Pharmaceutical Sciences, Shenzhen Campus of Sun Yat-Sen University, Shenzhen, 518107 China; 2 School of Pharmaceutical Sciences, Dali University, Dali, 671000 China

**Keywords:** Alzheimer’s disease, Stem cell technology, Disease modeling, Pathogenesis, Neuron–glia interaction, Stem cell-derived therapy

## Abstract

**Background:**

Alzheimer’s disease (AD) is a prevalent form of dementia leading to memory loss, reduced cognitive and linguistic abilities, and decreased self-care. Current AD treatments aim to relieve symptoms and slow disease progression, but a cure is elusive due to limited understanding of the underlying disease mechanisms.

**Main content:**

Stem cell technology has the potential to revolutionize AD research. With the ability to self-renew and differentiate into various cell types, stem cells are valuable tools for disease modeling, drug screening, and cell therapy. Recent advances have broadened our understanding beyond the deposition of amyloidβ (Aβ) or tau proteins in AD to encompass risk genes, immune system disorders, and neuron–glia mis-communication, relying heavily on stem cell-derived disease models. These stem cell-based models (e.g., organoids and microfluidic chips) simulate in vivo pathological processes with extraordinary spatial and temporal resolution. Stem cell technologies have the potential to alleviate AD pathology through various pathways, including immunomodulation, replacement of damaged neurons, and neurotrophic support. In recent years, transplantation of glial cells like oligodendrocytes and the infusion of exosomes have become hot research topics.

**Conclusion:**

Although stem cell-based models and therapies for AD face several challenges, such as extended culture time and low differentiation efficiency, they still show considerable potential for AD treatment and are likely to become preferred tools for AD research.

## Introduction

Alzheimer’s disease (AD) is a common, chronic neurodegenerative disorder characterized by the extensive distribution of neuronal tangles and amyloid plaques in the brain along with astrogliosis, neuroinflammation, synaptotoxicity, neuronal loss, and vascular alterations [28]. In recent decades, AD has been a subject of intensive research, however, no cure has been developed to date due to a limited understanding of AD pathogenesis. Fortunately, stem cells—as a category of cells with multi-directional differentiation potential—present new and powerful tools for AD disease modeling, drug screening, and cell therapy.

**Fig. 1:**
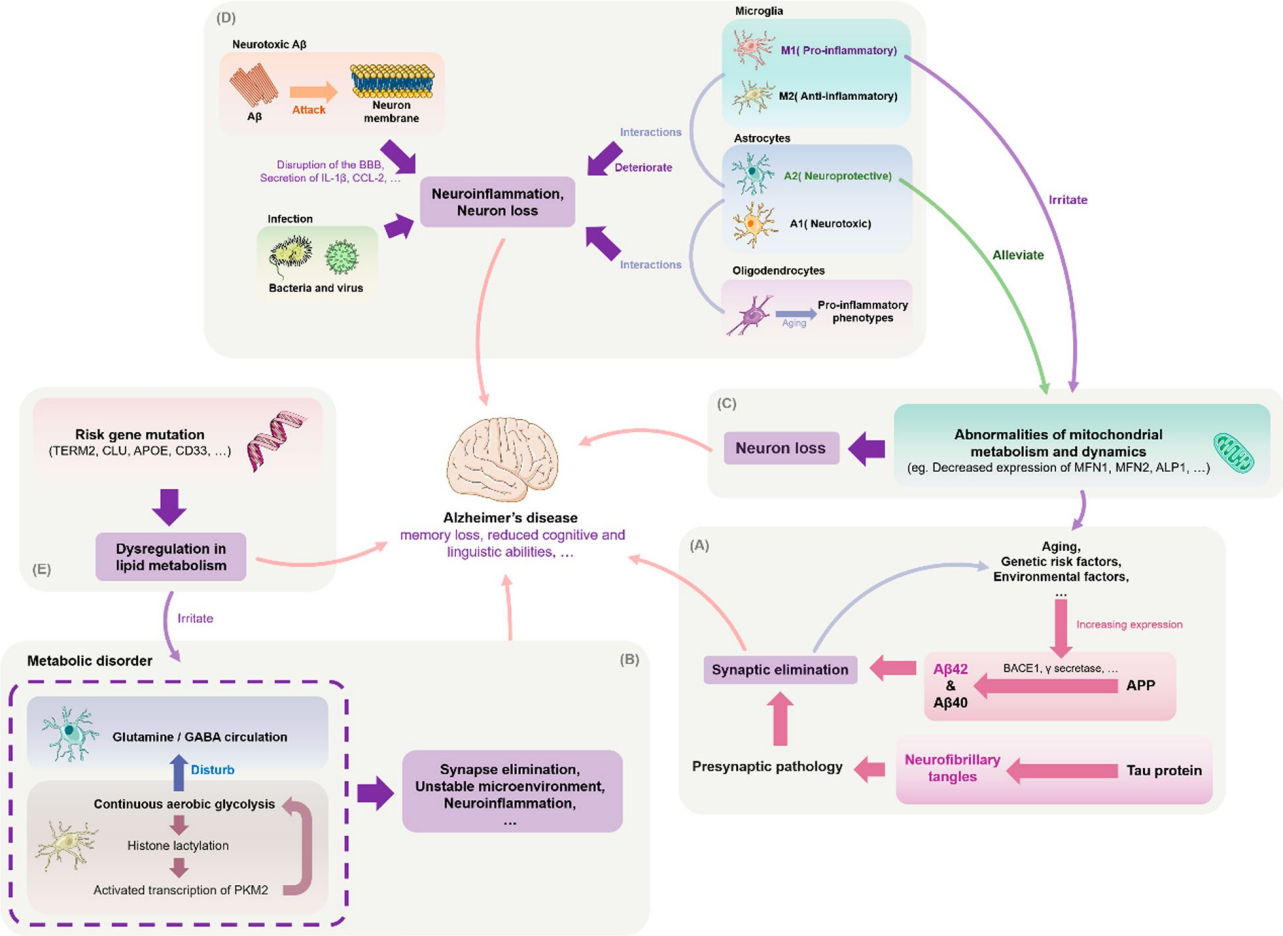
Possible pathogenesis of Alzheimer's disease. **A** Amyloid-beta precursor protein (APP) is cleaved by BACE1 and other enzymes in the amyloid pathogenic pathway to form different isoforms of Aβ (this process can also be affected by gene mutations), which rapidly aggregate to form neurotoxic oligomers that can induce synaptic loss and further cause cognitive impairment and dementia. Simultaneously, Aβ can contribute to tau pathology by increasing abnormal phosphorylation and the formation of toxic neurofibrillary tangles (NFTs), which can lead to neurodegeneration. According to the synaptic defect hypothesis, synaptic damage is the underlying cause of AD and defective synapses may lead to the formation of neurotoxic Aβ. **B** One potential mechanism of AD progression is dysregulation of multiple metabolic pathways. Microglia glucose metabolism disruption promotes a vicious cycle of "glycolysis-histone lactation-PKM2", leading to an imbalance of microglia homeostasis and neuroinflammation. Astrocyte metabolic failure also impairs the neurotransmitter glutamate/GABA glutamine cycle and causes synaptic dysfunction. **C** Dysfunctional mitochondrial dynamics can result in aberrant mitochondrial fusion fission, in turn causing synaptic degeneration and neuronal injury. Such dynamics include diminished expression of the proteins that control this process. By transferring healthy mitochondria, astrocytes could reduce the negative effects of damaged mitochondria on neurons. This function is blocked in chronic inflammatory situations, which may hasten AD progression. The removal of Aβ and emergence of tau disease may also be impacted by aberrant mitochondrial autophagy. **D** Microglia, astrocytes, and oligodendrocytes have pro-inflammatory phenotypes that promote neuroinflammation and the development of AD. The three types of glial cells interact with one another to speed up neuroinflammation. For example, activated microglia secrete IL-1a, TNF-a, and C1q, which cause reactive astrocytes to develop into the A1 neurotoxic phenotype, whereas oligodendrocytes cause astrocytes to develop into the NF-B signaling or A1 phenotype. Other harmful processes in AD include the attack of Aβ on its own neuronal cell membranes and bacterial-viral infection. **E** Mutations in common risk genes, including APOE, triggering receptor expressed on myeloid cells 2 (TREM2), CLU, and CD33, may disrupt normal functions—particularly lipid metabolism in glial cells—thereby increasing the risk of AD development

The conventional animal models used to study AD, which is mainly constructed through transgenesis or induced aging, have considerable limitations due to the complexity of the human cerebellum, such as neuronal sub-type changes [45]. Stem cell-based models show significant potential for solving these problems. By reprogramming donor somatic cells with disease morphology, one can obtain induced pluripotent stem cells (iPSCs) with the ability to expand and differentiate, thereby freeing researchers from reliance on animal models. Moreover, the ability to culture 3D iPSC-based organoids and construct humanized AD models has enabled researchers to explore other possible factors related to pathogenesis, such as immune system disorders, impaired synapses, abnormal mitochondrial structure and function, and mutations in risk genes (Figure 1). As the amyloid hypothesis continues to be investigated [99], understanding the contributions of these pathological phenomena to disease etiology and neuronal death is of great importance for understanding AD pathogenesis and developing new therapies.

The development of stem cell modeling technology has also launched a new era of personalized pharmaceuticals. Current drug screening methods are expensive, time-consuming, and complicated. In contrast, stem cell-based models can accurately simulate pathological changes in the AD brain, providing more possibilities for AD-related drug screening. Using stem cell technology, we can understand the effects of drugs at the earliest stage of analytical development and testing and make accurate assessments of their side effects and efficacy.

Currently, Aβ and tau pathologies remain the focus of new therapies undergoing clinical trials for AD, which include monoclonal antibodies targeting Aβ[50, 88, 109, 131], the β-secretase (BACE1) inhibitor that inhibits secretase activity to lessen Aβ deposition [35], and drugs that target tau protein [94]. Unfortunately, most clinical trials have not achieved good results and some have been terminated due to serious side effects [193]. Such cases demonstrate the challenges of sub-cellular therapies while highlighting the advantages of stem cell therapies, such as the possibility of reversing the entire pathological process of AD [136, 142]. Transplantation of stem cells in animal models has been shown to provide significant benefits by affecting multiple pathological mechanisms and ultimately improving cognitive function [103]. Targeting ganglion stem cells in the body to activate and generate new glial cells or neurons is another strategy for stem cell therapy. New targets have been recently identified in animal models to produce these pro-regenerative effects [77].

However, the heterogeneity of stem cells and their survival and migration rates after transplantation limit their applications. Researchers have attempted to address these issues through methods like modifying stem cell encapsulation, pretreatment, or co-delivery. Alternatively, direct use of stem cell-derived exosomes for AD treatment may achieve better results than various complex stem cell modification methods [128], as they show significantly improved pathology and cognitive function in preclinical experiments. Their therapeutic potential may be even further exploited after engineering. For example, the production of exosomes can be increased by 3D culture or co-culture with chloroquine, NH4Cl, and other molecules. Also, by changing the content of oxygen, hydrogen ions, and nutrients in stem cell culture medium, the release of exosomes can be altered, however, this may require exploration through further experiments. Fusion of protein sequences or peptides with exosome membranes can also be used to build devices for targeted delivery, and electroporation is the most widely used method for membrane fusion [181].

In this review, we summarize progress in stem cell-derived AD models and the principles and applications of stem cells and derived therapies to alleviate AD pathology. We then discuss the prospects of stem cells as models for further investigation of AD pathogenesis and drug screening, as well as application prospects and development directions for stem cells and derived therapies targeting AD.

## Stem cell-derived disease models in AD research

Stem cells are a category of cells with the potential of self-renewal and multidirectional differentiation. They originate from various tissues such as placenta, adipose tissue, and dental pulp and have numerous potentially useful functions such as tissue and organ regeneration and treatment of diseases (especially severe diseases).

One crucial application of stem cells in AD research is the creation of patient-derived human cellular models. Examples of these are organoids and assembled spheroids, which are multicellular models composed of different brain cell types. These models can be used to test the impact of genetic, chemical, and environmental factors on AD, as well as to evaluate new genetic engineering and genome editing techniques to unravel disease pathogenesis. Another area where stem cells are used is the development of in vivo models that more accurately reflect human physiology and disease, such as humanized animal models. As transplanted stem cells or their derived tissues/organs retain their original features and functions, humanized models constructed by transplanting stem cells of human origin into animals can circumvent ethical constraints associated with human experimentation [164]. Utilizing information on AD-related risk genes, biological processes and pathways, and biomarkers provided by advanced multi-omics technologies, such as single-cell and spatial transcriptomics, long-range sequencing, next-generation proteomics, and other high-throughput cellular phenotyping platforms, stem-cell-derived disease models can provide comprehensive insights into the pathogenesis, progression, and drug screening of AD [182, 185, 186, 194, 196, 199].

### 2D culture systems

The in vitro model of human iPSCs was first established in 2007 in the laboratory of Prof. Shinya Yamanaka [125]. More than a decade later, iPSCs have been differentiated into various pathogenic cell types including neurons and glial cells to mimic central nervous system (CNS) developmental processes and profile disease phenotypes. To promote the differentiation of iPSCs into the above cell types in 2D culture and prohibit their differentiation to mesoderm or endoderm, dual TGF-β (SMAD) inhibition is utilized to generate neural stem cells (NSCs) with higher purity [19]. By inhibiting the expression of sonic hedgehog (SHH) signaling through enhanced Wnt signaling, [66, 129], NSCs can develop into cortical neurons. Cortical neurons, such as cortical glutamatergic neurons, are mainly distributed in the neocortex and hippocampus, which are early affected tissues in AD [78]. By further manipulating extrinsic signaling conditions, NSCs can differentiate into other disease-related cell types such as cholinergic neurons [11]. Substantial loss of cholinergic neurons has been observed in the brains of early-stage AD patients. After prolonged culture, neurogenic cultures can undergo gliogenesis conversion to produce various glial cells [18, 42]. Dysfunction of such glial cells is also considered a cause of AD pathology. As a result, it is increasingly important to study interactions between various neuronal subtypes and glial cells using such models. Moreover, compared to using human-derived stem cells of limited origin or rodent cell lines from widely varying species, 2D co-culture systems using iPSCs yield highly scalable cell models that enable rapid exploration of cellular pathophysiology, including transcriptional, genetic, and signaling pathways [136, 142].

### 3D culture systems

To solve the problem of simulating AD pathology in vivo, 3D neuronal culture technology, has been developed based on 2D cell culture systems. Unlike traditional 2D cultures, 3D neuronal cultures better simulate the natural environment for cell survival in organisms, thereby achieving cell–cell interactions and more realistic biochemical and physiological responses. To date, many rigid scaffolds have been developed to support neuronal culture, including super-porous/non-porous hydrogels, polydimethylsiloxane with micrometric cavities [14], sintered titanium [70], and hydrogels or matrigels [59]. 3D soft matrix scaffolds can also be constructed using hydrogels, fibronectin-bound differentiated iPSCs, and 3D printing techniques. Not only do these approaches maintain the electrophysiological activity of neural tissue compared to rigid scaffolds, but they also allow the mimics to be made into different shapes as needed to accommodate multiple platform applications.

Although scaffolds can somewhat better mimic the developing brain, they still lack the cellular diversity, structural complexity, and physical structure visible in vivo [5]. The development of 3D organoid models and microfluidic organoids has effectively addressed some of these deficiencies. 3D organoids are grown from iPSCs generated from human stem cells or adult cells and have a similar composition and structure as primary tissues as well as ease of manipulation and cryopreservation. Organoids have been shown to have well-defined radial glial cells, astrocytes, and neurons that better mimic human cortical structure during development or in disease states [22, 102, 170]. Early brain organoids derived from directed neuroectodermal differentiation did not contain mesodermal lineage cells, such as myeloid microglia or endothelial cells [213]. However, this can now be achieved by co-culturing human umbilical vein endothelial cells (HUVECs) and microglia-like cells isolated from primary tissues or differentiated from human embryonic stem cells (hESCs) with brain organoids [209]. Functional microglia can also be directly generated from modified unguided protocols [201] or hESCs in human cortical organoids (hCOs) [167]. The multiple types of human brain cells contained in the above organoids play an important role in neurodevelopment and the occurrence of neurodegenerative diseases [206]. As a result, 3D organoids can be used to study human diseases that are difficult to simulate using animal models, such as AD.

Microfluidic organ-on-a-chip technology, which is a “human-on-a-chip” cell culture system for studying physiological processes, can be used to grow neurons, glial cells, endothelial cells, and skeletal muscle cells while providing a highly fluidic extracellular environment and maintaining fluid isolation with a multi-lumen device to better outline organ-like structures [47]. This approach also allows one to manipulate biological specimens and cells and simulate pathological conditions with extraordinary spatial and temporal resolution to reveal mechanistic insights into the disease, thereby accelerating drug discovery, screening, and toxicology research [118, 159].

### Humanized animal models

Humanized animals are animals carrying functional human genes, cells, tissues and/or organs, immune system, or microorganisms that are used in biomedical research. As they can fully simulate the pathological phenotypes and disease mechanisms of human AD, humanized AD animal models play an important role in AD research. There have been recent developments of genetically humanized AD animal models. For example, Pang et al. [202] successfully developed a gene knock-in rat model capable of fully simulating AD and more accurately simulating AD pathogenesis in the brain. Andersen et al. [162] used CRISPR-Cas9-based gene editing to develop a haploid insufficiency model of sortilin-related receptor 1 (SORL1) (which encodes the endosomal recycling receptor) in Gögenting’s miniature pigs, providing support for studying the pathological characteristics of AD caused by *SORL1* haploinsufficiency. However, at present, the use of stem cell transplantation technology to establish cellular humanized animal models—also known as chimeras—is mainly applied in the research fields of immunology autoimmunity, transplantation, infectious diseases, and tumors, and is not widely used to study neurodegenerative diseases like AD. To create a humanized AD model, [16] transplanted cortical neurons produced from human iPSCs into the brains of transgenic Tg (APP/PS1-21) mice. Their goal was to reveal species-specific vulnerability of human neurons to AD pathology. Disease processes and treatment advancements in AD have been clarified by this model. Another humanized AD model was constructed by Espuny-Camacho et al. [177], who transplanted healthy human neural precursor cells (NPCs) from iPSCs into the frontal cortex of immunodeficient neonatal transgenic APP/PS1-21 mice [177]. Their study revealed significant degeneration of transplanted healthy human neurons upon exposure to Aβ, microglia, and astrocytes [101]. Chimeric models based on stem cell technology can offer further insights into AD pathogenesis. For example, by constructing a chimeric mouse model with grafted Clusterin (CLU)^C^ (CLU rs11136000^C^ mutation) medial ganglionic eminence progenitors (CLU hiMGEs), Chen et al. clarified that over-activation of the sphingolipid pathway may be the underlying mechanism of CLU AD. Using CLU^C^ humanized mice, the study shed light on AD pathogenesis, and highlighted the need for more research on GABA transmitter synthesis abnormalities [169].

**Fig. 2:**
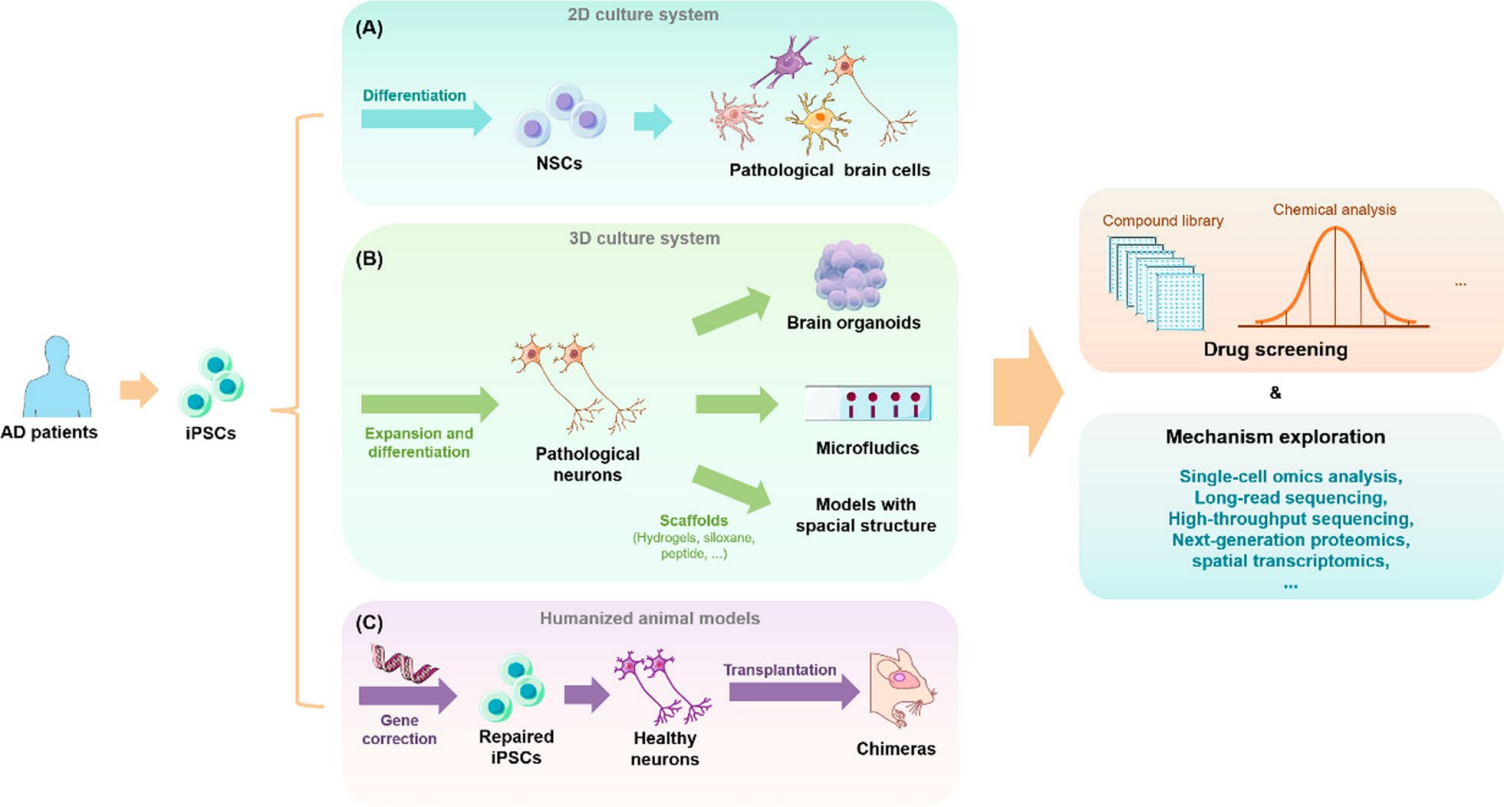
Stem cell-derived disease models in AD research. **A** iPSCs obtained from AD patients are used in 2D stem cell culturing systems to generate pathological neurons for drug screening and mechanism exploration. **B** Stem cell 3D culture system: using iPSC from AD patients to construct organoid and microfluidic organ-on-a-chip models for drug screening and mechanism exploration. **C** iPSCs from AD patients with gene alterations resulting in the production of normal neurons, which were then used to create a humanized animal model

Humanized AD animal models constructed by the stem cell transplantation technique described above have significant advantages over 3D models. Most importantly, they provide longer latency to capture more critical AD neuropathological features including astrocyte proliferation, microglia proliferation, synaptic deficiency, dendritic degeneration, neuronal cytoskeleton abnormalities, progressive 4R tau expression, and tau hyperphosphorylation [16]. These advantages allow researchers to understand the relative timing of the appearance of different pathological features of AD, thus providing a valuable platform for studying how aging causes or affects neurodegeneration (Fig. 2).

## Contributions of stem cell technology to AD research and treatment

Great progress has been made in iPSCs in neural differentiation in recent years, and there are now many protocols for inducing or reprogramming stem cells to differentiate into different types of cells and neurons involved in AD pathogenesis, such as neural progenitor cells [9], microglia [1], astrocytes [134], oligodendrocytes [138], endothelial pericytes [3], glutamatergic, aminobutyric acidergic neurons [23, 41], and basal forebrain neurons [90], resulting in the possibility to create physiologically relevant in vitro models and more promising means of AD treatment. In the following sections, the value and potential of stem cells and their derived 2D, 3D, and humanized animal models in AD mechanism research, drug screening, and cell therapy are further explored.

### Stem cell-derived platforms for studying AD pathogenesis

With the rapid development and application of the above-described stem cell-derived disease models, understanding of AD has improved. The emergence of these new models has helped scientists study the pathological mechanisms of AD from different perspectives, such as “risk genes”, “immunity”, and “mitochondria”, and propose new pathological hypotheses of AD while improving classic hypotheses.

#### Amyloid hypothesis

In 1991, John Hardy and David Alsop proposed the β-amyloid cascade hypothesis [180], which suggested that extracellular β-amyloid (Aβ) in the cerebral cortex is the central component of brain senile plaques in AD patients and that its deposition is the central cause of patient morbidity. Aβ is a short neurotoxic peptide produced by successive cleavage of APP by β-secretase and γ-secretase enzymes [115]. It can rapidly aggregate to form oligomers that are recognized as potent synaptotoxins, inducing synaptic loss and memory impairment to some extent [135]. In contrast, autosomal dominant mutations in APP, presenilin-1 (PSEN1), and presenilin-2 (PSEN2) have been observed in patients with familial AD (fAD), and these mutations are responsible for the pathogenic aggregation of Aβ peptides into neuritis plaques [130]. Even without such mutations, similar pathological changes can be observed in sporadic AD (sAD). Thus, it is presumed that insufficient brain Aβ clearance is the key event leading to its aggregation [84].

Stem cell-derived disease models have played a key role in the formation and development of the above hypothesis. In 2011, Yagi et al. established the first fAD-iPSC-derived neuron model carrying *PSEN1* and *PSEN2* mutations. These neurons were found to have increased Aβ42 secretion, while *PSEN* fAD mutations impaired γ-secretase activity, thus confirming the pathogenic link between fAD and PS1 and PS2 mutations [149]. To further identify the phenotypes common to fAD, [116] selected cortical neurons produced by iPSCs carrying different *APP* and/or *PSEN1* mutations as a model. They found that endosomal transport defects in AD were caused by APP cleavage by-product β C-terminal fragment (β-CTF), as shown by analysis of their transcriptome and translatome. This finding suggests that the pathological mechanism of AD is not limited to Aβ, and that future research should target other APP metabolites (such as β-CTF) to prevent neurodegeneration [116]. Using this disease model, future research could also test whether endosomal changes are related to neuronal death and whether blocking the accumulation of other APP metabolites such as β-CTF can prevent neurodegeneration. It is worth mentioning that humanized AD mouse models have also played a key role in evaluating the amyloid hypothesis [208].

In addition to the 2D models described above, 3D cell culture systems and humanized AD models have been used to assess this hypothesis. Li et al. [190] constructed a 3D hydrogel culture system and observed many extracellular Aβ deposits in fAD ReN cells after 6 weeks of differentiation. Choi, Park, and Lee [171] established a 3D neurospheroid model consisting of prenatal rat (not human) cortical neurons and synthetic Aβ42 to test Aβ toxicity in a microfluidic platform [171]. In 2017, researchers generated a humanized AD model by transplanting human iPSC-derived cortical neurons into the brains of transgenic Tg (APP/PS1-21) mice [16]. In the comparison of human and mouse neurons, it was found that the Aβ plaque morphology in human neuron clusters was more diffuse than the dense core plaques in mouse brains, further revealing the species-specific vulnerability of human neurons to AD pathology. This model has provided key insights for the development of the AD amyloid hypothesis.

#### Hyperphosphorylation of tau protein

Given the failure of amyloid-based therapies for AD, researchers’ interest in tau has increased. Tau is a protein with roles in the stabilization and assembly of neuronal microtubules (MAP) that is mainly expressed in neurons, oligodendrocytes, and astrocytes in the CNS and peripheral nervous system [127]. Tau proteins control microtubule stability through heterodimerization and phosphorylation. Hyperphosphorylation of tau leads to abnormal function and aggregation, further forming neurofibrillary tangles (NFTs). The combination of toxicity acquired by aggregates or their precursors and the deleterious effects of loss of normal tau function in disease states may be an important cause of neurodegeneration [8].

Scientists are currently attempting to use stem cell-derived pathological models to address an important but unresolved question in AD: the relationship between tau pathology and Aβ, i.e., whether tau protein aggregation is secondary to Aβ deposition or an independent factor directly contributing to neurodegeneration. In 2012, Israel et al. [53] reprogrammed primary fibroblasts from two patients with fAD and found that tau phosphorylation was increased in neurons carrying APP repeat mutations and that β-secretase inhibitors significantly reduced tau phosphorylation by blocking Aβ production [53]. Wang et al. [137, 215, 216] found that the elevated level of hyperphosphorylated tau in iPSC-derived neurons carrying apolipoprotein E4 (APOE4) was not dependent on Aβ in *APOE4*, paving the way for studies on other factors underlying tau phosphorylation. A more extensive study was conducted by [121]. who performed shRNA knockdown screening of more than 50 AD risk genes in iPSC-derived neurons to examine the effects on Aβ deposition and tau hyperphosphorylation and their interconnections. They found that FERM domain containing kindlin 2 (FERMT2) expression in neurons promoted late-onset AD (LOAD) formation and deterioration by increasing Aβ accumulation and tau levels [121].

Taken together, these findings demonstrate that human iPSC-derived neurons can effectively reflect the early features of AD pathogenesis, including the accumulation of pathogenic Aβ species and early tau disease. These results have important implications for further research and refinement of hypotheses and interrelationships between tau pathology and Aβ. Notably, both 3D culture models and humanized organoid models better show extracellular β-amyloid aggregation and NFT pathology than 2D neuronal cultures, providing a more robust model for AD research [121].

#### Risk gene mutations

Genome-wide association studies (GWAS) have identified many risk genes associated with different types of AD. Examples of these risk genes are summarized in Table 1.

**Table 1:** This table divides AD into two common types (EOAD and LOAD) and lists their common risk genes and mutation ways respectively

AD type	Gene	Mutation type	Mechanism	Supplement	
EOAD/fAD [163]	APP	Val717Ile/London mutation	increase in Aβ42/Aβ40 ratio and total Tau	autosomal-dominant	
	PSEN1	missense mutations, small insertions, deletions, and genomic deletions	interfere with the fusion of the γ-secretase complex	PSEN2 carriers may onset later than PSEN1 carriers on an average level	Complete penetrance
	PSEN2	Missense mutations			incomplete penetrance
LOAD	APOE [179]	Over 44 risk loci	See below for details		
	TREM2 [225]	R47H mutation	See below for details		
	CD33 [187]	rs3865444 and rs12459419 risk variant	inhibit of cellular activity and of functions	N/A	
	INPP5D [168]	missense Mutations	Increase plaque-associated microglia	N/A	
	SORL1 [173]	Point mutation	Alter APP trafficking at the cell surface	Parkinsonian disease (PD) features	

Stem cell technology combined with gene editing techniques can shed light on the role of risk genes in AD and the interplay between environmental factors and genetic background. CRISPR-Cas9-based genome editing and its derivatives have enabled efficient genetic modification of hiPSCs. The resulting “homozygous pairs” have greatly improved the resolution of disease-specific phenotypes and circumvented the need to collect patient cell lines with different genetic backgrounds, thereby facilitating research on the role of risk genes in AD [37].

Apolipoprotein E (APOE) is the strongest genetic risk site for LOAD [25].It also plays an important role in maintaining membrane homeostasis, supporting synaptic integrity, and healing brain injury. In humans, APOE has three isoforms: APOE2, APOE3, and APOE4. [158] demonstrated that human iPSC-derived astrocytes are a powerful platform for exploring the role of APOE ε4 isoforms in AD. They found that, when co-cultured with iPSC-derived neurons, human iPSC-derived APOE ε4/ε4 astrocytes promoted neuronal survival and synaptogenesis, whereas APOE ε4/ε4 astrocytes were not as effective as astrocytes with the APOE ε3/ε3 genotype in supporting these neurotrophic functions [158]. Sienski et al. (2021) also used this platform to explore the relationship between lipid metabolism and APOEε4 mutations. They demonstrated that APOE4, but not APOE3, disrupts the lipidome of iPSC-derived astrocytes [113]. 3D brain organoids from AD patients have been used in related studies. Zhao et al. [223] found that brain organoids from AD patients carrying APOE ε4/ε4 showed greater apoptosis and lower synaptic integrity and that APOE4-related pathways were directly associated with pathological tau protein accumulation. CRISPR/Cas9 technology has also contributed greatly to research on APOE4. For example, using CRISPR/Cas9 and gene expression analysis, Lin et al. observed that the APOE4 variant affects a series of genes that regulate lipid metabolism and transport and that the effect is cell-specific. These findings provide a reference for the design of future gene therapies targeting AD risk genes [68].

*TREM2*, another important AD risk gene, has been extensively studied using stem cell-derived disease models. *TREM2* encodes the myeloid trigger receptor 2, which is a cell surface transmembrane protein produced in the brain by microglia that primarily regulates microglial cell survival and activation [75]. Mutations in this gene increase the chance of developing AD by 200–400% above the normal level [43, 61]. McQuade et al. [195] analyzed microglia differentiated in CRISPR-modified TREM2 knockdown iPSC lines [195]. By combining multi-omics analysis with a chimeric AD mouse model, they found that *TREM2* deletion reduced microglia survival, impaired phagocytosis of key substrates including APOE, and inhibited SDF-1α/CXCR4-mediated chemotaxis, ultimately leading to impaired uptake of β-amyloid plaques in vivo. By combining chronic demyelination paradigms and cell sorting with RNA sequencing and lipidomics, Nugent et al. [200] elucidated the causal link between abnormal cholesterol transport and metabolism in TREM2-deficient microglia and the accumulation of toxic proteins in the brain, confirming that TREM2 deficiency exacerbates AD pathology.

In summary, stem cell technology can also be used to conduct GWAS. The genomes of iPSCs are more stable and reflect the genetic information of individuals more realistically than the traditional cell lines used in GWAS research. Furthermore, since iPSCs can differentiate into various cell types associated with AD, they can better mimic GWAS-related diseases. CRISPR-based gene editing technology can introduce knockouts or single-nucleotide polymorphism (SNPs)-targeted mutations into iPSCs, thus providing a high-throughput platform to functionally validate AD-related SNPs and genes [46].

#### Abnormal mitochondrial structure and function

There is growing evidence that impaired mitochondrial biogenesis and function are involved in many neurodegenerative diseases and can impair synaptic neurotransmission, which is essential for normal cognitive function. As a neurodegenerative disease [114], aging is a major risk factor for AD. However, the reprogramming step of iPSC technology resets age-related features (e.g., mitochondrial dysfunction, telomere shortening, etc.) back to the fetal stage, [74], meaning that stem cell models are useful for not only studying disease mechanisms but also disease processes—especially early biological and pathological events that disrupt neurological maintenance. Stem cells and their derived models thus provide a highly exploitable platform to study structural-functional changes in mitochondria early in AD development. They can also help us better understand the molecular basis of mitochondrial heterogeneity in structure and function. Stem cells with normal or abnormal mitochondria can be generated from healthy people and patients and then differentiated into various cell lineages. This approach allows us to investigate how the state of mitochondria affects stem cells’ ability to generate different cell types and regulate remodeling in disease-specific cells. As proof of concept, we can use patient-derived stem cell platforms to investigate the role of mitochondria in AD development. This approach represents a paradigm shift in understanding the role of mitochondria in human disease and opens up new avenues for AD research and therapy.

Using stem cell-derived models, many studies have shown that mitochondrial dysfunction, including metabolic disorders, increased ROS production, altered mitochondrial morphology, and dysregulation of mitochondrial quality control (QC) [211, 218], are early signs of AD progression and contribute to disease progression. GWAS have identified variants in the ABCA7 gene encoding ATP-binding cassette (ABC) subfamily A member 7 that are strong risk factors for late-onset AD [183]. By constructing ABCA7-deficient iPSC-derived cortical organoids, Kawatani et al. proposed a new mechanism of AD-related neuronal injury caused by ABCA7 function loss: ABCA7 deficiency interferes with mitochondrial lipid metabolism, damages mitochondrial respiration, produces excessive ROS, enlarges mitochondrial morphology, and leads to disruption of mitochondrial homeostasis and synaptic function of neurons [188]. Mitochondrial QC includes fission and fusion processes, mitochondrial trafficking, and mitophagy [214]. The constant change of mitochondrial structure and shape through continuous fusion and fission is a process called mitochondrial dynamics [165]. There is growing evidence that dysfunction of mitochondrial dynamics may be the pathological cause of delayed onset neurodegenerative diseases such as AD [165]. When the contents and organelles of mitochondria are damaged, mitochondrial autophagy is triggered.[191]. Mitochondrial autophagy is a process that selectively removes damaged mitochondria, plays an essential role in regulating the number of intracellular mitochondria and maintaining normal mitochondrial function [161]. In the early course of AD, Fang et al. [178] observed widespread impaired mitochondrial autophagy in iPSC human AD neurons.[178] To investigate the effect of APP cleavage products on mitochondria function, Lee et al. (2022) used induced neural stem cells (iNSC) derived from fibroblasts of an AD patient origin as a model. They concluded that the accumulation of APP-derived C-terminal fragments (APP-CTFs) within the structural domain of mitochondria may be responsible for dysfunction such as impaired mitochondrial autophagy [64]. Martin-Maestro et al. [80] observed that dysfunctional mitochondria accumulated due to impaired mitochondrial autophagy in skin fibroblast iPSC-derived neurons carrying *fAD*-associated mutations. They further determined that the defective autophagic process was caused by a reduced autophagic degradation phase due to lysosomal abnormalities [80]. They also constructed hiPSC-derived NSCs carrying the fAD-associated PS1 M146L mutation and identified mitochondrial respiratory chain defects, such as low expression levels of key enzymes like cytochrome c oxidase (complex IV), cytochrome c reductase (complex III), fusion proteins like mitofusin-2 (Mfn2) and optic atrophy 1 (OPA1), and autophagy-associated proteins light chain 3 (LC3)**.** Reduced expression of lysosomal associated membrane protein 1 (LAMP1) and transcription factor EB (TFEB) may further contribute to the accumulation of defective mitochondria. These findings suggest that early metabolic alterations and impaired mitochondrial QC may have consequences for the pathophysiology of AD.

Because autopsy does not provide relevant information about disease progression, it is critical to develop model systems to study early molecular changes. AD human iPSC-derived models are thus complementary tools to study the degenerative process. Researchers have used such models to demonstrate the critical role of early events like mitochondrial dysfunction and impaired mitochondrial autophagy on AD pathogenesis [81]. Besides DNA in nuclei, cDNA mutations in mitochondria are another potentially important cause of AD, as well as the root of aging, and thus deserve our attention [222]. Researchers should focus on mitochondria signal pathways and mitochondria-targeted therapy to improve obstacle stress and energy metabolism in AD, ultimately improving our understanding of the interaction between genetic and environmental factors that contribute to mitochondria dysfunction in AD.

#### Immune system disorders

There is an intricate link between the immune system and CNS; when this balance is broken, it can cause neuroinflammation and, ultimately, AD. Neuroinflammation is an inflammatory response in the CNS caused by various pathological injuries. In this process, CNS glial cells, such as microglia and astrocytes, produce various pro-inflammatory cytokines, such as Interleukin-1β (IL-1β), IL-6, IL-18, tumor necrosis factor (TNF), and chemokines, as well as small molecule messengers and reactive oxygen species [31]. CNS glial cells thus play an important role in the progression of inflammation.

Stem cell technology allows researchers to study the interplay between immune function and AD using models like co-culture of immune cells from a single patient with their organoids [82]. As stem cell-derived 3D cultures can generate human oligodendrocyte spheroids, astrocyte spheroids, and neurons, these models can be applied to investigate glial cell functional abnormalities, different glial cell interactions, neuronal interaction networks, and the mechanisms of AD immune disorders. Bianco et al. [10] used a microfluidic platform to investigate the complex roles of different regional astrocytes in neuroinflammation. They inoculated astrocytes from the cortex and hippocampus, as well as primary hippocampal neuron cell types, from rat embryos in different chambers of the microfluidic network. By examining the effects on neuronal viability in two neuroinflammatory injury models (i.e., metabolic stress and exposure to amyloid β protofibrils), they clarified the differential contributions of brain region-specific neuroglia in two in vitro models. This study reflects the superiority of 3D microfluidic organ chips with multiple chambers to independently control culture conditions and selectively differentiate stem cells into different cell types with different stimuli. As this is also an open-ended method, it allows close monitoring of the specific contribution of each cell type by analyzing morphology, viability, calcium kinetic, and electrophysiological parameters when different chambers are micro fluidically connected [10]. Such models can reveal the complexity of cell–cell interactions in neuroinflammation, which is of great importance for elucidating the molecular mechanisms involved. To study the effect of the immune system on neuronal signaling, Guttikonda et al. [44] established a well-defined human pluripotent stem cells (hPSC)-derived tri-culture system containing pure populations of hPSC-derived microglia, astrocytes, and neurons. Using this culture system, they were able to demonstrate that increased complement C3 production in the brain is partly caused by microglia activation and its interaction with astrocytes, which in turn worsens neuroinflammation [44].

Synthetic models with AD features have also been developed. For example, Park et al. [95] used this technique to create a human ternary culture model containing NPC-derived neurons and astrocytes as well as immortalized human microglia. This method reflected the phenomena of microglia recruitment, neurotoxic activity, and nitrogen monoxide (NO) release damaging AD neurons and astrocytes and is suitable for further investigating the complex molecular mechanisms of neuroinflammation that underlie AD pathology [95]. Human pluripotent stem cell-derived brain-like Organoids co-cultured with immune cells from AD patients can also be used to better understand inter-glial interactions and provide an ideal experimental platform to explore the mechanisms of AD immune dysfunction under an in vivo growth environment.

As a proof of concept, we can use patient-derived stem cell platforms to study the role of the immune system and various glial cell communication disorders in the development of AD. This represents a paradigm shift in understanding the role of immunity in human disease and opens new avenues for AD research and treatment. In the future, new models could be developed to evaluate novel therapeutical interventions for AD such as immunomodulatory therapy, anti-inflammatory drugs, and immunoglobulin therapy.

In summary, reprogramming somatic cells from AD patients back to stem cells that can be re-differentiated into in vitro disease models to probe disease progression and pathogenesis provides a new approach for understanding the pathogenesis of human neurodegenerative diseases. This approach also takes advantage of the reverse engineering platform to monitor and determine key pathogenetic events during disease development. The hallmark of degeneration of the aging brain is selective cell vulnerability in disease-specific patterns many decades after the birth of long-lived brain cells. The traditional paradigm for investigating neurodegeneration has been to define the biological processes and pathways mediating brain cell dysfunction and death during the adult life; neurodegenerative diseases have thus been considered a discrete pathological entity rather than a continuum of a common pathogenic process. The new perspective considers neurodegenerative diseases as a fundamental disorder of neural development in which subtle impairment in the regional programming of neural development results in selective neural and brain network vulnerability to a spectrum of late-life stressors. Indeed, emerging data support the possibility of pathogenic associations between abnormalities in the birth and death of vulnerable brain cell subtypes [210].

### Development of stem cell technology platforms for drug screening

Good disease models can be used to explore the diverse pathogenesis of diseases and support drug screening and development. However, drugs to stop AD progression are currently lacking. One important reason for this is that current drug screening methods typically rely on holistic animal models and cell lines as pharmacological and toxicological testing systems. Animal models of AD have species differences and cannot fully represent human responses, while most human cell lines are immortalized tumor cell lines that hardly reflect the reactivity of normal human cells.

Alternatively, in vitro differentiation of stem cells as a model of normal human development can be used for drug screening or to obtain multiple normal somatic cell types to test the toxicological effects of drugs on specific cells. The use of iPSC in vitro differentiated cells to screen new drugs can greatly reduce the number of experimental animals required for drug experiments. The use of stem cells to establish models that comprehensively reflect the pathological phenotype and disease mechanisms of human AD is thus of great significance for AD drug development and screening.

In 2017, Kondo et al. presented a platform for drug discovery and development using human iPSCs and compound screening. They used 13 iPSC-differentiated pure cortical neurons from AD patients. After two rounds of screening, the final screen q yielded six potential compounds that could minimize Aβ40 and Aβ42 levels. Such models can help overcome the drawbacks encountered in drug development and screening using standard human iPSC platforms [56, 123].

Simple models of iPSC-derived neurons have failed to account for interactions with vascular cells, glial cells, and the blood-brain barrier (BBB). The BBB is a major factor limiting the action of AD drugs. Moreover, BBB dysfunction and catabolism have been observed in AD, which in turn triggers a series of inflammatory responses and neuronal loss and neurodegeneration. The development of specific AD BBB models and their application in preclinical studies are therefore important for improving the success of AD drug clinical trials. Many recent studies have reported successfully applying combined iPSC and microfluidic technology to mimic the BBB in a neuro-neuroglial interaction model [132]. The use of iPSC-derived BBB models in combination with gene editing (e.g., CRISPR/Cas-9 technology) to mimic disease-associated mutations or introduce artificial mutations (e.g., gene knockout) can better reflect altered brain dynamics in disease states and help screen new drugs that can reach deeper neural tissues through the BBB [4, 112]. Gene-edited iPSCs expanded and differentiated in vitro into different cell types can also be transplanted into AD animal models to generate human-animal chimeras for drug screening [136, 142].

### Potential of stem cell-based therapies for AD

**Fig. 3:**
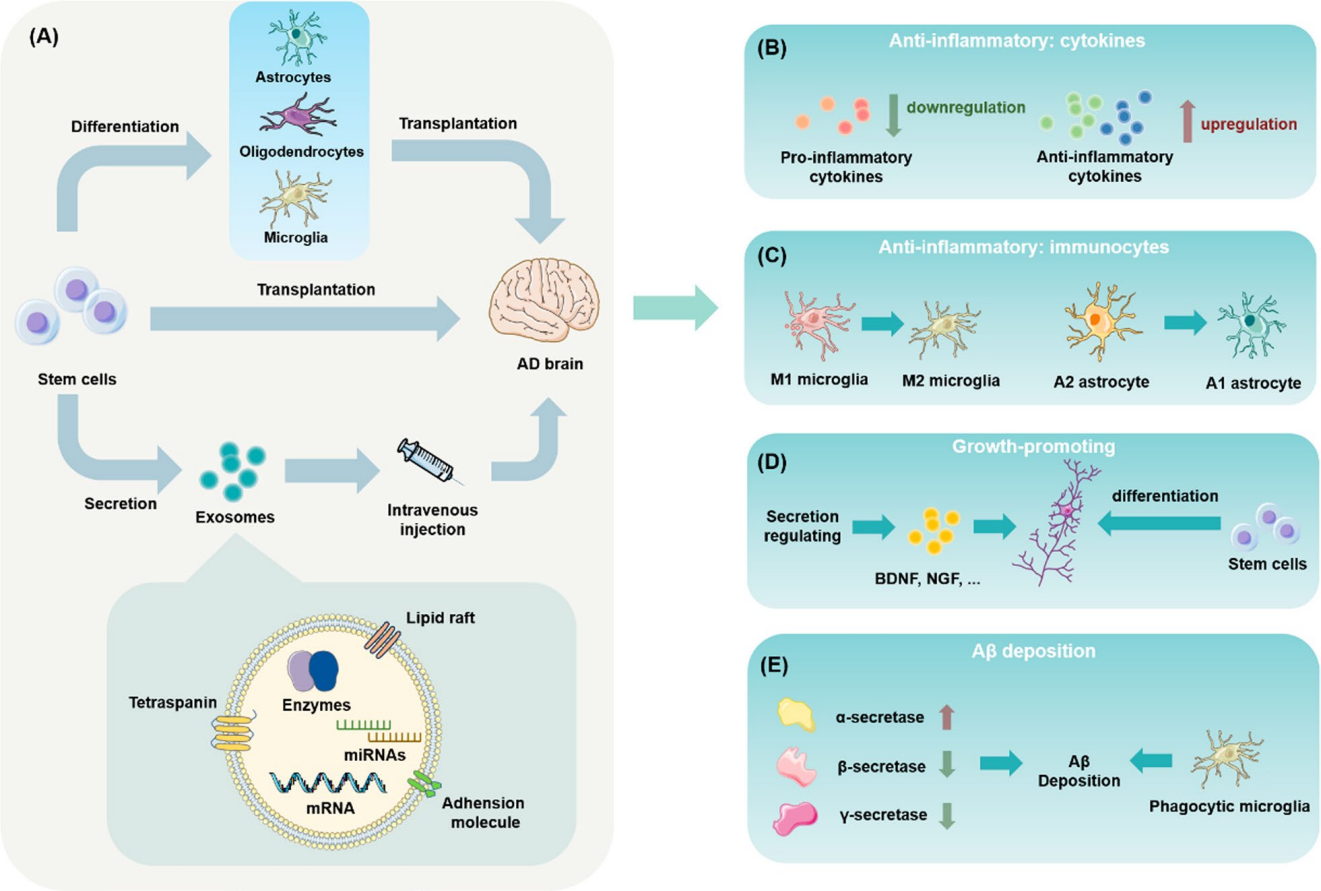
The role of stem cell-based therapies in the treatment of AD. **A** Through direct transplantation, retransplantation following differentiation into neural cells, and secretory exosome infusion, stem cells offer various benefits for treating AD. **B** Stem cells with their differentiated cells and derived exosomes regulate the release of a range of cytokines, reducing levels of pro-inflammatory cytokines (IL-1β, IL-6, TNF-α) and increasing levels of anti-inflammatory cytokines (IL-1 receptor antagonist (IL-1ra), IL-10) in cerebrospinal fluid. **C** The phenotypes of microglia and astrocytes are changed by stem cells through their differentiated cells and exosomes, shifting them from a pro-inflammatory to anti-inflammatory phenotype and decreasing the inflammatory response in the brain and promoting neuroactivity. **D** Stem cells with their differentiated cells and derived exosomes are upregulated with Brain-Derived Neurotrophic Factor (BDNF), Nerve Growth Factor (NGF), etc. The stem cells transplanted through exogenous pathways are integrated into the neural network to regenerate synapses. **E** As MSCs with their differentiated cells and exosomes activate microglia, they specifically phagocytose Aβ or regulate the expression levels of Aβ-related enzymes (e.g., upregulate α-secretase activity or downregulate β-secretase activity) to clear existing Aβ deposits

In addition to their value as disease models and for drug screening, stem cells have played a significant role in treating AD. The transplantation of stem cells to treat AD is mainly based on their ability to differentiate into neurons and glial cells that can replace damaged cells, release cytokines to activate endogenous neurogenesis, and regulate immune cell status. Transplanted stem cells also can be induced to differentiate into glial cells or corresponding precursor cells to improve the neuronal environment by repairing the functions of supporting cells, ultimately repairing overall neural network function. Stem cells can also act therapeutically through the contents of their secretomes, e.g. exosomes. Unlike monoclonal antibodies or enzyme inhibitors that target Aβ to slow down the pathological process, stem cells have the potential to rebuild the integrity of the CNS. Moreover, given the interconnectedness of the various mechanisms of AD pathology, targeting just one for treatment may not be as effective as therapies targeting various factors—stem cells have the potential to treat AD via multiple mechanisms (Fig. 3).

#### Stem cell transplantation therapy

The formation of Aβ and its damage to the neurological system is one of the best known pathological processes in AD [141]. Clearing Aβ from the brain is thus thought to be one of the most promising ways to treat AD. Stem cell transplantation could reduce Aβ deposition through the following mechanisms: activation of microglia to eliminate Aβ through specific phagocytosis [62, 63], regulation of the expression levels of enzymes related to Aβ synthesis or degradation (e.g., upregulation of α-secretase activity or downregulation of γ-secretase or β-secretase activity) [224], or removal of existing Aβ deposits through immunotherapy. These effects on Aβ could occur separately or simultaneously [189]. The clearance of Aβ is frequently accompanied by microglia, however, this does not mean that microglia activation is essential for stem cell transplantation therapy to achieve Aβ clearance [144].

Hyperphosphorylation of tau protein and NFT production are also significant pathological aspects of AD, and different tau structures may mediate different pathogenic processes. However, more extensive research on how stem cells can improve tau pathology is required [36, 38]. Nevertheless, it is important to investigate potential improvement in tau pathology by stem cell therapy given the current assumption that tau pathology may develop independently of Aβ pathology. There is adequate evidence that stem cell transplantation can improve the activity of cells damaged by abnormal tau protein neurotoxicity. For example, Zilka et al. [226] co-cultured AT tau cells, a model of AD cells developed from human truncated tau protein (Tau151-391, 4R), with mesenchymal stem cells (MSCs) and found that this greatly enhanced cell viability, demonstrating that MSC therapy rescued cells from toxic tau protein. The researchers suggested that this effect of MSCs is partly attributed to the complex bioactive components in their secretomes. They also reported that MSCs encouraged neurite development in cells expressing truncated tau. These findings demonstrate that stem cell therapy can reduce cellular damage caused by toxic tau proteins—at least in in vitro experiments [226]. However, the therapeutic effect of stem cell transplantation on tau-oligomers-burdened models still needs to be explored, for tau oligomers is now observed to cause serious synapse elimination [212].

One of the most significant therapeutic functions of stem cells in neurodegenerative disorders is replacement of senescent, damaged neurons. When stem cells like iPSC-NPCs are pre-induced in vitro to differentiate into cholinergic neurons and then reimplanted they could improve memory and cognitive function. They have been proven to survive and differentiate into cholinergic and GABAergic neurons when directly transplanted to lesion sites. The positive effects of stem cells on synaptic regeneration can be described as endogenous or exogenous: exogenous synaptic regeneration is realized through the integration of stem cells implanted into neural networks, whereas endogenous synaptic regeneration is realized through the promotion of synaptic regeneration via upregulation of factors positively associated with neurogenesis, e.g., BDNF, NGF, etc.

Many experiments have demonstrated enhancement of synaptic integrity and elevation of neuronal activity after injection of stem cells, and Basal forebrain-like cholinergic neurons (BFCN) progenitor cells are frequently utilized in experiments [184, 221], indicating the possibility of rebuilding neuronal circuits utilizing exogenous neural stem cells.

As an alternative to exogenous implantation, numerous studies have sought to achieve brain repair by promoting the expression of endogenous growth factors and, subsequently, repairing neuronal circuits. In many studies, the upregulation of proteins associated with antioxidation, synaptic connectivity, and neurogenesis such as sirtuin1 (Sirt1), BDNF, synuclein (SYN), and β-nerve growth factor (β-NGF), has been observed. [157, 172, 175] Stem cells’ secretomes or the trophic factors produced after their differentiation into glial cells also act as sources of neurotrophic factors. For instance, using co-culture experiments, Peng et al. [203] showed that BM-MSCs can differentiate into Schwann cells and promote the quantity and length of sprouting neurons of dorsal root ganglia neurons.

However, other studies suggest that the delivery of neurotrophic factors is not the primary benefit of stem cells for treating neurodegenerative illnesses [34]. Although delivery of neurotrophic factors may appear to be less effective at rescuing neurodegeneration, this technique can be utilized to enhance the therapeutic efficacy of stem cell transplantation.

For example, using BDNF-transfected MSCs for transplantation, Song et al. [117] showed that the neuroprotective effect of BDNF increased the activity and synaptic integrity of primary cultured neurons in APP/PS1 mice. The transplanted BDNF-MSCs differentiated into neuronal cells, increased the number of local cholinergic neurons, promoted Acetylcholine (Ach) secretion, and improved cognitive function [117]. Overall, synaptic regeneration may be more effectively promoted by combining the secretion of neurotrophic factors with the exogenous replacement of neurons.

Given increasing emphasis and support for the “infection theory” of AD, neuroinflammation mitigation has become an innovative treatment approach [51, 85], and the modulatory effects of stem cell infusion therapies on immunological function in the CNS are becoming focal areas.

As one of the most common immune cell types in the CNS, microglia maintain and protect neural cells by controlling inflammation and preserving the integrity of the microenvironment, among other methods [166]. In AD, related gene expression in certain microglia changes during the course of the disease [83], the expression of these genes in certain microglia has been shown to rescue neurodegeneration [61]. Thus, altering microglia activity is considered a potential method for treating AD. In APP/PS1 mice treated with human umbilical cord blood-derived mesenchymal stem cells (hUCB-MSCs), [63] demonstrated an increase in microglia in the alternative activation state and a decrease in neuroinflammation. In addition, Yokokawa [220] showed that MSCs transplantation into the brains of APdE9 mice induced microglia to convert from the M1 phenotype to the M2 phenotype and engage in phagocytosis, while also reducing neuroinflammation and enhancing the redox state. Besides, the transition between different microglia phenotypes, lower expression of pro-inflammatory factors (TNF-, IL-6, and Monocyte chemoattractant protein-1 (MCP-1)) and decreases in the quantity and volume of microglia in the cortex have been observed in stem cell transplantation studies [197]. 

Rather than a one-way influence, there is mutual action between neural stem cells and microglia [207]. Thus, the efficacy of stem cell transplantation is likely to be influenced by the microglia’s state of activation. In support of this, a study by Popova et al. [204] showed that microglia could induce transcriptional changes in neural stem cells and reduce interferon signaling response genes, while interferon-induced transmembrane protein 3 (IFITM3) was previously shown to upregulate γ-secretase activity to increase Aβ production. By co-culturing activated microglia with NPC, Lilienberg et al. [67] demonstrated that naïve microglia do not affect the proliferation of NPCs and enhanced neurosynaptic growth. Using this co-culture system, the authors also observed that the nitro blebbistatin-triggered synaptic development was enhanced by naïve microglia. [67]. Popova et al. [204] showed that microglia can induce transcriptional changes in neural stem cells and accelerate the emergence of synchronized oscillatory network activity in brain organoids, which has implications for the integration of stem cells into the original neural network following transplantation. These findings have revealed the potential of using drug induction in stem cell therapy and the important effect the environment’s microglia status has on neural stem cells. More importantly, not all instances of activated microglia cause damage to neurons. Thus, pre-regulating the state of microglia in the environment or combining it with drug induction may need to be considered when designing stem cell transplantation therapies.

In terms of other parts of the immunoregulation system is the CNS, the glymphatic system, which is related to various diseases like Parkinson’s [122], diabetes [55], hypertension [89], and aging [57], has recently attracted attention for its role in brain homeostasis. The glymphatic system controls the clearance of various toxic compounds that develop with aging or other changes from the brain, including Aβ proteins linked to AD. Astrocytes play a significant role in the glymphatic system, such as removing waste proteins by regulating cerebrospinal fluid shunting via aquaporin-4 (AQP-4). A promising approach to treating AD thus involves altering astrocyte activity through stem cell transplantation.

Previous studies have reported that stem cells can inhibit the proliferation of astrocytes and encourage the development of non-reactive astrocytes, which have the capacity to support synaptic regeneration, heal injured neurons, and engage in phagocytosis and Aβ catabolism [6]. Because abnormal cerebrospinal fluid-mediated immune function has significant implications for AD pathology and stem cell transplantation has been shown to improve some causes of glymphatic system disorders—including upregulation of aquaporin (AQP-4) expression and attenuation of astrocyte proliferation—the beneficial effects of stem cells on the lymphoid-like system warrant further investigation [97, 143]. One novel avenue for future stem cell research in AD may be stem cell manipulation of astrocyte protein expression, which in turn influences the effectiveness of material exchange and waste clearance in the lymphoid system.

In summary, stem cell transplantation therapy has been shown to not only improve typical pathology indicators of AD, such as Aβ and Tau fiber tangles, but also to have a reciprocal modulatory effect on numerous important immune system cell types, including microglia and astrocytes. These various efficacies most likely work together to achieve the neuronal regeneration and cognitive improvement observed after transplantation. Pre-conditioning the microenvironment of the treated area or combining neural stem cells with certain glial precursor cells (GPCs) may be a workable therapeutic approach when considering the interactions between cells and their environments.

#### Stem cell-derived glial cell therapy and models

Although neurons may be directly responsible for cognitive function, their sustained activity relies largely on support from glial cells, which account for the majority of brain cell populations. The repeated failures of strategies to eliminate abnormal protein aggregates in AD patients—such as Aβ aggregation inhibitors, Aβ antigens, anti-Aβ monoclonal/polyclonal antibodies, γ-secretase inhibitors, γ-secretase modulators, and β-site amyloid precursor protein cleaving enzyme (BACE) inhibitors—may teach us a lesson: although glial cells have long been ignored, they make important contributions to the onset and progression of AD.

Glial cells are now widely recognized to have potential for AD research and therapy. Using disease models based on glial cells, it is possible to investigate AD pathogenesis in a more systemic way. With rapid developments in stem cell technology, more attention is being paid to glial cell transplantation therapies. The three major approaches to transplanting glial cells derived from stem cells are microglia transplantation, astrocyte transplantation, and oligodendrocyte transplantation. NSCs, GPCs, and pluripotent stem cells (PSCs) are some sources of stem cells used for glial cell transplantation. GPCs can also be obtained from PSCs. Given the potential of stem cell-derived neuroglia platforms and therapies, it is necessary to discuss possible approaches to glia-based platforms and transplantation therapy.

##### Stem cell-derived glia in disease modeling

Given the important role of glial cells in maintaining a stable brain environment and neuronal health, it is necessary to involve multiple glial cell types in the construction of disease models. Accordingly, glial cell-based models of AD need to be developed using different glia types to establish in vitro and in vivo models. Such models will enable researchers to study AD in a more controlled and systemic manner to identify novel treatments.

Many studies using AD disease models containing glial cells have provided new understandings of pathological processes. Complement C3 is a protein that is increased under inflammatory conditions and implicated in synaptic loss. Guttikonda et al. [44] identified the main reason for increased C3 in AD and the main cell types involved being microglia versus astrocytes using a hPSC-derived microglia and tri-culture system model [44]. By using neurons, astrocytes, and microglia in a 3D microfluidic platform, Park et al. [95]. presented a 3D human AD tri-culture model that mirrors microglial recruitment and important neurotoxic activities in AD [95].

To summarize, to deepen our understanding of AD pathogenesis and promote AD therapy, more research is needed to help design glia-based AD models that mirror typical activity in AD. A tri-culture system comprising all three kinds of neuroglia may be a more promising approach than using a single glial cell type. This suggests that human brain organoids comprising neurons and several types of glial cells should be an ideal platform for AD research. However, additional studies about the different glia subtypes are warranted, since the subtypes have different functions and may thus have different effects on AD pathology.

##### Stem cell-derived glia in AD therapy

Traditional brain cell therapies tend to use ectogenic neurons to replace damaged ones. However, this type of transplantation has numerous drawbacks, many of which are caused by neurons’ ability to be excited. In some cases, neuron transplantation can cause convulsion in mice, which can eventually lead to death. Ectogenic neurons are thought to be responsible for these severe side effect because they are more excitable [105]. Further research is clearly needed to explore additional cell types for cell replacement therapies for AD. Glial cells, including microglia, astrocytes, and oligodendrocytes, play crucial roles in maintaining the environment in the human brain and regulating processes over the lifecycle of neurons. Glial cells are now considered a promising choice because they positively influence neurons in many ways, including secretion of factors, phagocytosis, and direct interaction between cells. Glial cells are also comparatively safe for transplantation because they are non-excitable, thereby protecting the recipient from hypo-excitation.

**Microglia transplantation for AD**

Due to their ability to activate and escalate neuroinflammatory responses, microglia have long been considered one of the main contributors to damage caused by neuroinflammation. However, based on in-depth studies on microglia function and state, current microglia therapies no longer advocate comprehensive suppression of their functions, but rather distinct strategies to promote alleviation of neuroinflammation that are tailored to the microenvironment of the specific disease. Due to the extended longevity of microglia and the fact that the gradual loss of homeostasis is associated with AD pathogenesis, the low efficiency of nascent microglia in removing aggregates of toxic proteins may encourage the pathological processes of AD. To restore their natural immunological filtering function, senescent microglia should be rejuvenated or healthy microglia could be replenished endogenously or exogenously as part of an AD treatment strategy. Exogenous microglia supplementation or stem cell-derived microglia transplantation are potential approaches to reestablish central immune clearance. In bone marrow transplantation (BMT), microglia-like cells produced from iPSCs, ESCs, or monocytes are the main cell sources for microglia transplantation. Several studies have reported successful transplantation of microglia into the mouse brain and validation of functional integration of microglia after transplantation [145, 145, 147, 147]. Notably, the microglia showed oligodendrocyte phagocytic and synaptic phagocytic functions characteristic of normal microglia. Microglia transplanted into mice brains are stimulated by Aβ to alter expression groups, i.e., gene expression that tends to be in an activated state [76]. However, proof of their mitigating effects on AD pathology is limited. It has also been shown that TREM2 expression is lower in hPSC-derived microglia [32]. Given that low TREM2 expression was thought to have a positive effect on maintaining microglia homeostasis, human pluripotent stem cell-derived microglia may help maintain a stable phenotype that is beneficial to remission of neuroinflammation after transplantation into the brain.

Although there is insufficient research on microglia transplantation to treat AD pathogenesis, transplanting microglia could theoretically help reduce harmful protein buildup from pre-existing, hard-to-remove senescent microglia and help carry out typical synaptic pruning and oligodendrocyte phagocytosis. Transplantation of a single microglia type (or equivalent precursor cell, such as a primitive macrophage progenitor cell (PMP) might not result in significant changes in AD pathology; transplantation combining several stem cell types might be preferable. Depending on the lesion location, the appropriate subtype of microglia can be selected and paired with astrocytes or neural stem cells to more accurately reflect the environment that supports neurons in vivo.

**Astrocyte transplantation**

The properties and activities of astrocytes are altered in many neurological illnesses, including AD, which may worsen the degenerative processes. It is well established that astrocytes can be generated from stem cells or histocytes. Transplanting astrocytes to achieve recovery of functions is regarded as one of the most promising directions for AD treatment.

At present, astrocyte transplantation therapy uses human embryonic stem cell (hESC) differentiated astrocytes, human iPSC differentiated astrocytes, human fetal brain-derived astrocytes, or human fetal spinal cord-derived astrocytes. The best source of astrocytes is currently differentiation using stem cells (e.g., iPSCs, hESCs), as it is difficult to achieve the quantity needed for transplantation therapy using astrocytes derived from the human fetal spinal cord and human fetal brain, which is crucial for efficacy [157].

The feasibility of transplanting astrocytes requires integration of glial cells into existing circuits. This was demonstrated by Zhang et al. [224] in their study showing that NSC-derived glial precursor cells could differentiate into astrocytes and integrate into existing circuits in the brain after transplantation, with a survival time of more than one year. Initially proposed to treat spinal cord damage, astrocyte transplantation has been proven to protect host spinal cord neurons, encourage axonal regeneration, reduce glutamate toxicity, and support restoration of motor, sensory, and respiratory functions [160].

The neurotrophic effects of astrocytes, microglia activation, and oligodendrocyte activation all support astrocyte transplantation for treating AD. Although there are only a few studies using astrocytes for AD therapy, the existing research provides preliminary proof that intracerebral transplanted astrocytes undergo morphological changes in response to Aβ deposition and that this morphology varies with AD stage [101]. It has also been demonstrated that human ESC-derived astrocytes stimulate the IL-4 signaling cascade by controlling the microglia phenotype and serving as neuroprotectants, which is consistent with previous findings on the clearance of Aβ by astroglia [98, 136, 142]. As demonstrated by increased AQP4 polarization facilitating deposit clearance and improved neuronal function [151], implantation of GPC-derived astrocytes was recently shown to result in long-term functional integration and sensory enhancement in elderly mice.

In addition to astrocyte transplantation alone, astrocyte transplantation has been proposed in combination with neuronal transplantation. Because astrocytes facilitate neuronal maturation and increase synaptic plasticity, combined cellular therapies may be advantageous for both maintaining astrocyte function and promoting neuronal differentiation [160].

**Oligodendrocyte transplantation**

Oligodendrocytes are the core cell type involved in myelination. They can be differentiated from oligodendrocyte precursor cells (OPC) capable of migration, expansion, and protecting axons and neurons. The degenerative process in AD frequently involves oligodendrocyte dysfunction, which can result in further neuronal loss and dysfunction. OPC transplantation has mainly been investigated for treatment of multiple sclerosis (MS). However, its potential for AD treatment should not be disregarded since OPCs play a crucial role in remyelination by producing new oligodendrocytes to replace damaged ones and recovering lost cells after injury. Additionally, the biphasic communication of OPCs with astrocytes makes their function closely related to that of the central immune system. Theoretically, OPCs can be transplanted alone to promote myelination and supporting axons, or transplanted with neurons to repair loss of functional cells in the brain.

Oligodendrocyte transplantation is less common and is typically intended to heal neurological impairment rather than to treat AD. For example, Windrem et al. [217] transplanted human glial precursor cells (hGPCs) for differentiation into oligodendrocytes and observed myelin repair in the brain of myelin-deficient shiverer mice; the researchers thought the cells would subsequently function as remyelinating oligodendrocytes and be normally recruited. This study proves that integration of transplanted GPCs differentiated into oligodendrocytes in the brain is possible. Notably, OPC transplantation may be a more durable approach to external regeneration of oligodendrocytes due to the potential of GPCs to differentiate into other glial cells.

#### Stem cell-derived exosome therapy

Although stem cell therapy has shown superiority over traditional drug therapy for AD, it still faces many problems. First, since stem cells have a low degree of differentiation and strong ability to divide, they are prone to tumors. Second, intranasal administration is often used to administer drugs to the brain, but is challenging with stem cells. Using MSCs (with a diameter of 30–60 μm) as an example, the large size of stem cells may block the pulmonary capillaries and induce immune rejection [87]. Thus, delivering transplanted stem cells to the brain is challenging and carries some risk [27, 96]. Autologous transplantation of MSCs is challenging in patients with fulminant disease due to the complex cell preparation process and time needed for cell transplantation [48].

Exosomes derived from stem cells have become a promising new cell-free strategy in the decades-long pursuit to improve the therapeutic efficacy of stem cells. Exosomes, as a part of stem cell secretomes, are diverse membrane-bound vesicles that function as intercellular messengers. They carry different loads including DNA, RNA, proteins, and lipids, among others. Stem cell-derived exosomes show molecular specificity due to engineering and drug accumulation and can induce potent action mediated by the activation of factors and molecules with variable concentrations and signaling cascades. These actions can be easily modified and enriched by engineering exosomes [24].

The physiological roles of extracellular vesicles (EVs), which are abundant in most body fluids and released by cells. In fact, transplanted stem cells function in the body mainly due to their paracrine effect. As important parts of the stem cell secretion group, the physiological functions of exosomes are similar to those of their stem cell counterparts [153].

Compared with stem cell transplantation, exosome therapy has low immunogenicity and a lower likelihood of forming tumors. Nanoparticles (NPs) between 10 and 1000 nm in size can encapsulate therapeutic molecules by targeting the transport process in the CNS and then effectively migrate to target organs after infusion without blocking pulmonary capillaries and causing immune rejection. Cell-secreted exosomes, which are lipid-membraned vesicles, can easily permeate the BBB to exert their physiological effects. Exosome therapy has also shown advantages for AD patients with acute onset.

Because stem cell-derived exosomes generally show comparable biological activity as stem cells and have many advantages, they have become a hot topic along with cell therapy in the treatment of neurodegenerative diseases—exosomes are believed to be able to clear Aβ protein in the brain, restore synaptic activity in the brain, reduce inflammatory responses in the brain, enhance neural network connectivity, and alter metabolism. They thus have great potential for treating a variety of diseases including AD.

Researchers in Taiwan [22] Chen, et al. recently established a mouse FAD model and showed that the level of Aβ in mouse cells after exosome treatment was significantly reduced. In this experiment, exosome therapy was also found to improve glucose metabolism in the brain and restore the protein-modifying enzyme histone deacetylase 4 (HDAC4), representing improvement in memory and cognitive function.

Some researchers have improved exosomes’ efficacy at reducing Aβ in the brain through combination with other drugs. [140] used BM-MSC-derived exosomes in combination with sphingosine kinase inhibitor (SKI-II) or sphingosine-1-phosphate-1 receptor blocker (VPC23019). They found that exosomes inhibited Aβ1-40, Aβ1-42, BACE1, and PS1 in AD mouse cortex and hippocampus, thereby improving spatial learning and memory.

Some studies have found that stem cell-derived exosomes have therapeutic effects on neurological function [73]. A proteomic analysis of brain tissue from AD mice treated with MSC-EVs showed that a variety of proteins in EVs exert neuroprotective, synaptic growth, and neurogenesis effects. In the above study, EVs were also found to have therapeutic effects on some risk genes that can cause AD. Compared with the control group, 1094 genes were upregulated and 267 genes were downregulated in the brains of AD mice after exosome treatment. Some of these upregulated genes—such as piccolo (PCLO), *TENM1*, and Neurite extension and migration factor (*NEXMIF*)—are positive for synaptic function and memory improvement, while some downregulated genes are associated with cell death, such as *BAD*. These results suggest that EVs can relieve nerve damage and significantly increase neurogenesis in AD mice.

Regarding inhibition of inflammation in the brain, controlling M1/M2 phenotypic switching of microglia is significant for treatment of AD. Exosomes derived from MSCs can convert from the M1 phenotype with a pro-inflammatory effect into the M2 phenotype with an anti-inflammatory effect, which can effectively reduce neuroinflammation and relieve AD symptoms [30, 71, 96, 119].

Many previous studies have used AD mouse models to prove the effect of exosomes on inhibiting inflammation. However, due to differences in molecular pathways and signaling functions between rodents and human microglia, a research group from Stanford University [40] chose to study the role of MSC-EVs in the human microglial cell line (HMC3). They showed that EV treatment reduced the expression of CD11b in cells and the number of proteolysis and exerted an anti-inflammatory effect by promoting M2 phenotypic transformation of microglia. Notably, these results are similar to findings in AD mice.

Recent studies have shown that stem cell-derived exosomes can act on astrocytes to reduce their activation, thereby reducing neuroinflammation and improving cognitive impairment in AD patients. The most intuitive use of stem cell-derived exosomes on astrocytes is to reduce the proportion of active astrocytes, which is manifested as reduced glial fibrillary acidic protein (GFAP) levels in the brain and colocalization of GFAP and amyloid plaques [22] [24, 137, 215, 216])

Some findings for stem cell-derived exosomes in AD therapy over the last five years are listed below (Table 2):

**Table 2:** Stem cell-derived exosomes for AD treatment

AD model	EV culture or drug delivery method	Time period	Results at the molecular level
Produce the FAD mutant overexpressed human APP using lentivirus in SH-SY5Y cells with K670N/M671L and V717I, and culture these cells in the Matrix gel culture model to obtain the human FAD neural cell model	In vitro* / *in vivo*:* intravenous injection	In vivo: 4 weeks	Brain-derived neurotrophic factor (Bdnf exon IV) and Homer1, as well as genes related to synaptic plasticity, such as glutamate receptor subunits GluR1, GluR2, NR2A and NR2B, and Syp levels were upregulated; The glucose metabolism and cognitive function were assessed in [18F] FDG-PET and NOR tests before and after MSC-derived-exosome treatment, respectively: MSC-exosomes restored glucose metabolism and improved cognitive function in whole brain regions; HDAC4 expression were downregulated [22]
SH-SY5Y cells transfected with the APPswe gene; APP / PS1Transgenic mice	Isolate exosomes using the 3D graphene scaffold as a substrate for human umbilical cord MSCs culture	14 days	Immunofluorescence analysis; upregulation of ADAM10 levels and downregulation of BACE1 levels; downregulation of microglia marker Iba-1 expression in the right hippocampus; diminished effects of TNF-α and IL1β [219]
generated offspring of female APP/PS1 mice crossed with WT male C57BL/6 mice	Tail vein injection	16 weeks( once every two weeks)	Significantly increased expression of SphK1 and S1P1; Morris water maze experiment: escape latency was significantly shorter in APP/PS1 + exosome group; significantly enhanced expression of NeuN in mouse cortex and hippocampus (DG area) [140]
9-month-old female APP/PS1 mice; 6-week-old male C57BL/6 mice	Intranasal drug delivery	2 weeks	Various proteins in EV including enkephalin, neuroplasmin and eIF5A promoted synaptic growth; upregulation of genes PCLO, TENM1, NEXMIF and deregulation of gene BAD [73]
Aβ administered bilaterally in the dentate gyrus of 7–8-week-old C57BL/6 mice		14 days and 28 days	Increased neurogenesis of SVZ; increased immune cell response [205]
APPswe/PS1dE9 AD mice	Intracortical injection	25 days/month	Decreased expression levels of Alix, AGO2, HSP70, TSG101, CD63 and CD9; decreased number of Smi31-32 positive spots around ThT-positive plaques [176]
C57BL/6 background heterozygous AβPPswe/PS1dE9 double transgenic mice	Tail vein injection	8 weeks(once every two weeks)	Increased levels of IDE and NEP, leading to Aβ degradation; decreased density of Iba-1-positive microglia; increased gene expression levels of YM-1, Arg-1, MRC1, FIZZ1 and CD163 (M1 microglia to M2 phenotype switch); significantly increased levels of Arg1 and Ym1 RNA; decreased levels of IL-1β and TNF-α, increased levels of IL-10 and TGF-β [30]
In vitro: cell model of phenotypic polarization toward M1 obtained by TNFα stimulation of microglia from C57BL/6 mice/In vivo: female triple-transgenic AD mice(3xTg-AD)	In vivo: Intranasal drug delivery	21 days	Decreased IL-6 secretion and increased IL-10 secretion; decreased expression of Iba-1 and CD68 [119]
SCI rats	Vein injection		Inhibition of nuclear translocation ofNFκB p65 subunit: decreased proportion of A1 astrocytes; decreased expression of GFAP and pro-inflammatory cytokines (TNF-α, IL-1α and IL-1β); decreased expression of Syn, MBP and NeuN [137]
5XFAD mice	EV obtained centrifugally from human bone marrow-derived MSCs in three-dimensional (3D) cell culture; Intranasal drug delivery	4 months	Slightly decreased GFAP levels; co-localization between GFAP and ThioS was significantly reduced[24]
In vitro: GFAP positive astrocytes	miR-146a transfected and non-transfected BM-MSC-derived exosomes		significantly reduced TRAF6 and NF-κB expression[198]
APP/PS1 Double transgenic mice B6C3-Tg	RVG-labeled MSC-Exo (improved targeting); Vein injection	4 months	GFAP expression was significantly reduced; TNF-α, IL-β and IL-6 expression was reduced, and IL-10, IL-4 and IL-13 levels were increased[174]
Streptozotocin(STZ)establishment of sporadic AD mouse model	Lateral ventricular drug delivery	5 days(daily continuous injection)	IL-1β, IL-6, TNF-α expression decreased; BDNF protein levels were upregulated[192]
APP/PS1 mice	Vein injection	2 weeks(once every two days)	Significantly decreased levels of iNOS mRNA and protein [210, 216]
In vitro: LPS-stimulated human microglia clone 3(HMC3)cells		24 h	CD11b expression; significant decreased level of iNOS; upregulated secretion of IL-8 or IL-6 (pro-inflammatory results) [40]

Researchers used different in vivo/in vitro AD models with different exosome delivery modalities, employed different treatment regimens and a range of molecular biology tools, found that stem cell-derived exosomes could degrade Aβ in the brain, restore synaptic activity, enhance neurotrophic factors, and reduce inflammation in the brain. Thus it acts as a treatment for AD

## Challenges and strategies for stem cell technology in AD research and treatment

(Fig. 4).

**Fig. 4:**
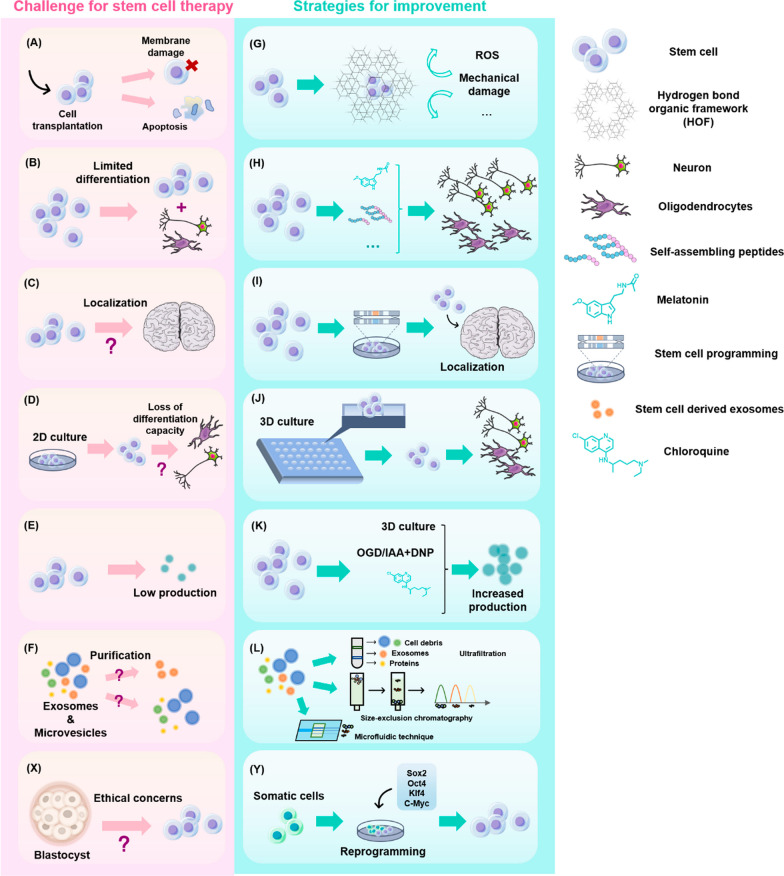
Examples of current challenges for stem cell-based AD therapies and strategies for improvement. **A** Bare stem cell transplantation results in challenges such as disruption of cell membranes and apoptosis. **B** Delivery after encapsulation with a protective organic framework protects stem cells from oxidative damage and mechanical damage. **C** Stem cell transplantation therapy may have limited efficacy due to low differentiation efficiency. **D** Co-culture of certain biomolecules with stem cells can enhance stem cell differentiation. **E** The efficiency of migration and localization of stem cells in the body after transplantation is difficult to guarantee. **F** Regulating the expression of specific genes can promote targeted migration of stem cells. **G** Traditional 2D culture methods may result in reduced or even lost differentiation potential of stem cells over time. **H** Beneficial characteristics, such as differentiation potential, can be better retained by using a 3D culture strategy. **I** Poor production of stem cell-derived exosomes. **J** Altered environmental conditions, such as oxygen–glucose deprivation, the addition of specific small molecules or 3D culture, can be effective for increasing exosome yields. **K** The extracted cellular secretion components are complex and it is difficult to isolate the therapeutic exosomes. **L** Exosomes of a certain particle size can be effectively separated using methods like ultrafiltration, volume exclusion method, microfluidics, etc. **X** Ethical concerns about the production of stem cells. **Y** The use of iPSCs for stem cell-based therapy

### Limitations and strategies of using stem cells in disease modeling and drug screening

Although human iPSCs have become a powerful tool for human disease modeling and have great potential for translational research in target discovery and drug development, various challenges remain. Human iPSC-derived neurons are sensitive and require extended culture time (80 days) to develop mature neuronal features; it is challenging to do this consistently and at a large scale by manually changing the culture medium [111]. Several strategies exist to overcome these challenges. Notch and γ-secretase inhibitors have been shown to shorten the maturation period of most neuronal cell types [13, 20]. Another approach includes overexpression of neuro-specific genes. For example, overexpression of *Ngn2* accelerates the generation of forebrain and motor neuron cells to within 10 days [52, 56]. In addition to shortening the time, the use of a high-throughput screening platform for aged neurons may be more translatable. Although scientists have differentiated stem cells into neurons, these neurons are functionally immature—similar to embryonic or early postnatal neurons. The limited maturity of neurons obtained using stem cell culture techniques diminishes their potential for neurodegeneration research. However, recent studies have found that differentiating human iPSCs into motor and cortical neurons and then culturing them on synthetic nanofiber coatings with high internal supramolecular motion can enrich them into more mature neurons [2]. Furthermore, since sAD is influenced not only by risk genes but also epigenetics, aging and environmental factors may contribute to its development. The ability of iPSC models to accurately mimic the sAD phenotype is another challenge in disease modeling and is thought to be an important reason why numerous AD therapies effective in preclinical trials have poor clinical outcomes [33]. The development of more effective sAD models is thus an important goal for the future.

Compared to 2D culture systems, 3D culture platforms are better suited to study AD pathophysiological mechanisms involving cell–cell interactions, controlled flow dynamics, circulating blood cells, and brain-specific microenvironments [100]. However, they still cannot mimic the intrinsic immune rejection reactions encountered in practical clinical applications. There are also many technical challenges facing organoid culture and microfluidic technology. For example, human brain organoids lack the vasculature of brain tissue in vivo, which plays a facilitating role for both nutritional supply and neuronal cell differentiation. This lack of vascularity leads to necrosis in brain-like organ centers, further interfering with normal development and neuronal migration routes. Although co-culture with vascular stem cells provides a partial solution to these problems, the issues of nutrient supply and the true blood microenvironment remain. hESCs expressing human ETS variant 2 (ETV2) have been used to successfully construct brain cortical organoids (hCOs) with vascular-like structures, including tight junctions, increased expression of nutrient transport proteins, and transendothelial resistance, thereby addressing the challenge of missing blood vessels [17]. However, further development is needed for their application in neurodegenerative disease. Regarding microfluidic technology, the requirements for reagent volume and reaction conditions are so stringent that small changes in the volume of reaction components can lead to changes in the results [118]. Therefore, the clinical usefulness of this method needs to be further explored.

For drug screening, it is extremely important to select the appropriate early-onset predominant phenotype as a marker to measure the effect of the tested drug. In AD, the dominance of tau hyperphosphorylation or Aβ aberrant deposition phenotypes has still not been elucidated. As a result, extensive mechanistic studies are needed before applying the findings to drug screening [21]. Since neurons, astrocytes, and microglia are highly specialized and disease-relevant cell types, the long time required for their differentiation limits the application of iPSC-derived glial cells in disease modeling and drug screening. The discovery of novel strategies to promote gliogenesis is thus crucial for large-scale drug screening. Novel gene editing tools and biomolecular technologies, such as single-cell RNA sequencing, could also enable more accurate disease modeling and drug screening processes.

To summarize, stem cells are widely used in disease modeling, drug screening, and cell therapy because of their unlimited self-renewal ability and potential to differentiate into all cell types. By using stem cell-based platforms, AD research can better study the fundamental biological mechanisms of the disease, develop new technologies, and innovate on therapeutic concepts. In the future, interdisciplinary collaborations should be fostered to build organoids or assemble spheroids and other multicellular models of human tissues comprising diverse cell types (e.g., excitatory/inhibitory neurons, astrocytes, oligodendrocytes, and microglia). This could enable rapid testing of genetic, chemical, and environmental perturbations to develop genetic engineering and genome editing tools to study the pathogenesis and treatment of the disease and develop in vivo models that more accurately recapitulate human physiology. We must develop multi-omics technologies, such as single cell and spatial transcriptomics, long read sequencing, next-generation proteomics, and other high-throughput cellular phenotyping platforms. We also need to develop software, data mining, and machine learning methods to dissect biological complexity in aging and disease and to perform functional and genomic analyses of multicellular models (e.g., bulk or single cell transcriptomic characterization, live cell imaging, and electrophysiology) and chemical biology-enabled multi-omics or chemical library-based drug screening studies.

### Limitations and strategies of stem cells and derived therapies

AD research that uses stem cell technology faces several significant challenges. These include: (1) limited capacity for self-renewal and differentiation of stem cells, (2) heterogeneity of stem cell populations and their derived cell types, and (3) ethical concerns surrounding the use of embryonic stem cells. However, ongoing efforts to overcome these limitations hold great promise for improving the quality and effectiveness of stem cell-based approaches. These efforts include: (1) the development of new techniques to promote differentiation, (2) preventing pre-mature death of stem cells and improving the therapeutic effect, and (3) investigating alternative, ethically acceptable sources of stem cells. Further research and innovation will therefore be critical to realize the full potential of stem cell technology in the fight against AD. The development and implementation of new strategies will improve the quality and utility of stem cell-based platforms for AD research and therapy, ultimately leading to better outcomes for patients with AD and related dementias.

#### Limitations of current stem cell therapies

##### Limited capacity for self-renewal and differentiation

The ability of stem cells to differentiate into specific cell types that carry out corresponding functions is frequently what determines how effective they are. As a result, inadequate differentiation rates or unexpected differentiation can significantly impede therapy. In certain cases, uncontrolled stem cell differentiation can potentially result in the development of tumors [12]. For these reasons, many studies using stem cells for therapeutic purposes pre-differentiate stem cells such as iPSCs into certain progenitor cells before transplantation [126]. Another factor that negatively affects stem cell therapy is the loss of beneficial properties that occur during processes such as culturing, including the differentiation potential of stem cells. The loss of these beneficial properties is partly due to traditional culture systems that do not accurately reflect the natural environment of the body. In response to this limitation, many studies aim to maximize the original properties of stem cells through methods like 3D culture or the addition of specific small molecules.

Large-scale clinical translation is also thought to be hampered by stem cells’ capacity for in vitro amplification. Since stem cells have a unique ability to differentiate, it is essential that they maintain their original characteristics during expansion. However, at present, additional generations of expansion are more suitable for small-scale operations in laboratories than large-scale clinical applications.

##### Heterogeneity of stem cell populations and their derived cell types

Other important factors affecting the efficacy of stem cells is heterogeneity [60] and derived cell types. Stem cell heterogeneity is universal,for example, NSCs contain different cell populations with both neurogenic and gliogenic potential [58]. MSCs are inherently heterogeneous cell populations, although the International Society for Cellular Therapy has clear restrictions on the types and proportions of molecules expressed on their cell surfaces. Explicit quantification of differentiation capacity and cell surface molecular expression serves to reduce the possible impact of stem cell heterogeneity on efficacy. It is well known that stem cells derived from different tissue sources can differ in the number and type of cytokines secreted, cell proliferation capacity, immunomodulatory capacity, etc. [39, 72, 120]. Stem cell heterogeneity is also related to tumorigenic potential. Notably, some studies have observed sustained proliferation and tumorigenic mass production after transplantation of hPSC neuronal derivatives in rodent models of PD [106, 107]. Stem cell heterogeneity must thus be accounted for in AD therapy research. [156] concluded that the ideal stem cell therapy for AD would transplant cells with different differentiation potentials precisely to the corresponding functional sites to achieve overall restoration of the functional network of the brain, which relies heavily on the identification and isolation of stem cell heterogeneity. [156] Besides stem cell heterogeneity, the heterogeneity of their derived cell types can greatly affect efficacy. For example, MSCs derived from healthy controls and patients can exhibit differences in certain functions. There are also unresolved questions whether MSCs of the same tissue origin have different epigenetic imprints. While current research on stem cells and heterogeneity of origin has focused on tumor therapy, we believe it is also valuable to consider this issue in the context of neural stem cell use for AD therapy. First, identifying and isolating subpopulations of neural stem cells in different states or with different differentiation potentials can both help determine the precise efficacy of specific subpopulations of stem cells and provide more possibilities for precise AD treatment. Second, emphasis on stem cell source heterogeneity could lead to better quality control of stem cells used for treatment, which in turn will provide assurance of efficacy.

##### Ethical and safety concerns surrounding the use of embryonic stem cells and cerebral organoids

Ethical issues have been widely discussed along with the development of stem cell technology, most of which are regarding the sources of stem cells. In the development of therapies using hESCs, the rationale of destroying early human embryos to treat diseases is controversial. Other current ethical issues concern the unlimited differentiation potential of iPSCs, which might be used for human cloning and thus the generation of human-animal chimeras and human embryos [133]. It is worth noting that human-animal chimeras are one of the most promising research platforms for AD pathology research. However, these models have been ethically questioned due to their similarity to the human brain. The crucial question is: does this technology have the potential to produce a human-like mind? Although the likelihood of human brain-like organs generating consciousness is currently considered minimal, we cannot exclude the possibility of future brain-like organs developing consciousness. To better understand AD pathology, we will likely need to develop more complex, organ-like models that more accurately reflect the human brain. [108] concluded that although there is currently no chance that cerebral organoids may develop typical consciousness, questions surrounding the presence or absence of consciousness may plague the development of brain-like organs. We thus need more precise protocols for the production, use, and destruction of different levels of organoids that consider ethical norms and safety.

At present, the primary source of ESCs is human embryos (blastocysts), which has limited the development of derivative therapies and poses considerable difficulties for clinical applications because of the difficulty of mass production. Although it seems promising to use iPSCs as an alternative source of ESCs, there are safety concerns because of the genomic instability of iPSCs—even with improved protocols for differentiation. As a result, before transplantation, many studies first promote in vitro differentiation into progenitor cells of specific neuronal types. The use of iPSC-derived gametes has also raised concerns about the potential exploitation of developed embryos, human nuclear transfer, and the risk of altering natural reproduction, including the potential to derive gametes for same-sex and asexual reproduction.

#### Strategies to overcome existing limitations and improve the quality of stem cell-based therapy and platforms

Several studies have sought to overcome the current limitations and improve the quality of stem cell-based therapies and platforms through methods like the development of new stem cell lines and new direct differentiation methods. The success of new encapsulation techniques, stem cell pretreatment, and co-delivery with small compounds also provides inspiration for further stem cell research.

##### Development of new techniques to promote differentiation

**Co-administration of small molecules to promote differentiation**

The process of differentiation in vivo*,* which is thought to play a significant role in efficacy, occurs in a variety of stem cell therapies, regardless of whether the cells are anticipated to differentiate into the target neuronal type in vivo or are infused after initial differentiation in vitro. The recent discovery that chiral small molecules promote stem cell differentiation in vivo has provided a new idea for improving the efficacy of stem cell therapy: co-administration of small molecules that encourage stem cell differentiation [110]. Observed that, when exposed to near-infrared light, nanoparticles with strong chirality can significantly speed up the differentiation of mouse NSCs into neurons. This finding suggests that strong chiral nanomaterials may be utilized to direct cell development and improve differentiation to achieve better efficacy in stem cell therapy. The use of near-infrared light might also lead to better control of the pro-differentiation effect [110]. 

Furthermore, [104] demonstrated that nanoparticle assemblies exerted circularly polarized light-dependent forces on the cytoskeleton and that light-induced periodic mechanical deformation of actin nanofibers at a frequency of 50 Hz stimulated neural stem cell differentiation into a neuronal phenotype. When implanted in the hippocampus of a mouse AD model, neural stem cells irradiated according to a polarity-optimized scheme substantially reduced the formation of amyloid plaques. These findings provide novel ideas for improving the efficacy of stem cells and a reference for the biological effects of circularly polarized light. Muñoz et al. [90] developed a differentiation scheme that successfully generated basal forebrain-like cholinergic neurons (BFCN) from iPSCs using small molecule inhibitors and growth factors. The study used LDN193189 and TGF-β inhibitors SB431542, FGF-2, SHH, FGF-8, BDNF, and NGF (separately or in combination) at different stages of differentiation to achieve a facilitative effect on the differentiation of iPSCs to BFCNs. These results also indicate that the strategy of adding small molecule inhibitors and growth factors at different periods has a regulatory effect on stem cell differentiation, providing theoretical support for promoting the differentiation of stem cells after transplantation. Since this experiment was conducted in vitro, further research is needed to determine whether the effects extend to intracerebral transplantation. We cannot rule out the possibility that the aforementioned differentiation-promoting small molecules could have unintended effects on the brain [90].

**Pretreatment of stem cells**

Since stem cells are sensitive to environmental changes, pretreatment is one of the most efficient strategies to change their phenotype and improve therapeutic efficiency. Stem cell transplantation can influence the number or type of surface receptors of certain types of somatic cells, enzyme expression levels, and secretome composition and have other effects that influence therapeutic efficacy by modifying environmental factors. Alternatively, small molecules can be added to the cell co-culture medium. It has been demonstrated that cytokines (such as IL-3, IL-6, HGF, etc.) increase the expression of CXCR4, which in turn encourages targeted migration of MSCs. Hypoxic preconditioning is another method frequently used to change the culture environment. In a study by [146], culture under hypoxic conditions induced an increase in SDF-1/CXCR4 signaling, which promoted MSCs migration. Similarly, [65] observed that hypoxia-induced MSCs had better viability, stronger proliferation potential, and better resistance to hypoxia and reactive oxygen species (ROS) stress. The proliferation and viability of neural stem cells have also been shown to be enhanced by hypoxia culture [15], which is a proven way to improve the survival rate of stem cells after transplantation.

Since transplanted neural stem cells must differentiate to be therapeutic, it is necessary to promote their differentiation at the lesion site. A study by [86] demonstrated that, after melatonin treatment with neurosphere culture, an inhibitory effect on NSC proliferation at a pharmacological concentration (25 μM) was observed: differentiation of NSCs into neurons—as well as oligodendrocytes—was promoted, while differentiation of NSCs into GFAP-expressing astrocytes was unaffected. Melatonin also enhanced the bioenergetic activity of mitochondria in NSCs and exhibited a dose-dependent pattern, suggesting that this stimulation method provides energetically pertinent support for the differentiation process. Melatonin therapy thus promotes NSC differentiation and neural replacement using NSCs combining pharmacological melatonin dosages is a potential regenerative therapy for neurological diseases. Studies on chemically created self-assembling peptides have also been performed to encourage NSC differentiation. [26] established a self-assembling peptide (DSP) that can spontaneously assemble into ordered nanofibers and contains a functional structural domain made of the laminin-derived amino acids Tyr-Ile-Gly-Ser-Arg (YIGSR); not only did this peptide increase cell viability in normal culture conditions, it also lowered the number of apoptotic cells induced by Aβ in vitro. NSC transplantation in AD rats treated with DSP was observed to enhance survival and neuronal differentiation of transplanted NSCs; increase secretion of anti-inflammatory and neurotrophic factors such as IL-10, brain-derived neurotrophic factor (BDNF), ciliary neurotrophic factor (CNTF), and insulin-like growth factor 1 (IGF-1); and reduce increased expression of pro-inflammatory factors such as TNF-α and IL-1β. These experimental findings are consistent with the hypothesis that the biomaterial DSP ameliorates AD pathophysiology by promoting NSC graft survival and development.

Brn-4 protein in MSCs also plays a positive role in treatment of AD, but miR-937 in stem cells hinders translation of Brn-4. Thus, administration of specially pretreated anti-miR-937 MSCs can contribute to the reduction and clearance of brain Aβ deposition [69].

**3D culture of stem cells**

Improving stem cell culture methods is another potential strategy to improve efficacy. Traditional in vitro 2D culture conditions may result in loss of stemness and other favorable qualities, as 2D culture mitigation does not accurately represent the ecological niche of stem cells in vivo. Supporting cells, extracellular matrix, and growth factors make up the microenvironment inhabited by stem cells in vivo. In contrast, 3D culture can better simulate intercellular and cell–matrix interactions. The natural environment of stem cells can be more accurately recreated in 3D culture and their favorable qualities can be maintained. Thus, one of the most promising methods currently being examined to support clinical translation of stem cells is 3D cultivation technologies. For example, Frith, Thomson, and Genever (2010) performed 3D culture of MSCs and demonstrated that dynamic 3D culture promoted MSCs to form aggregates with good cell viability. This effect was attributed to the combination of 3D culture with dynamic factors like increased cell–cell contact, which has been shown to improve stem cell subdifferentiation potential. Similarly, [139] designed culture substrates with micropatterns to culture MSC multicellular spheroids in 3D culture, finding that this method maintained cell function and improved differentiation potential in the longer term.

In conclusion, as 3D culture can better mimic the microenvironment of stem cells in vivo and help maintain stemness, it is of great value for advancing clinical translation of stem cell therapies.

##### Preventing premature death of stem cells and improving therapeutic effects

**Encapsulation of stem cells**

Many encapsulation approaches are designed to improve the low survival rate and prevent premature death of stem cells. Hydrogel encapsulation followed by medication administration is a common technique. Hydrogel materials are biocompatible, can be safely dissolved and absorbed in vivo, can remain stem cells aggregated and increase cell viability [92, 93]. Since MSCs have superior healing and anti-inflammatory potential when aggregated into spheres for administration, aggregation of stem cells may support efficacy [92, 93]. Multiple studies have shown that encapsulation with hydrogel materials improves the survival as well as the function of stem cells [49, 91].

However, after drug delivery, the volume of hydrogel molecules may restrict molecule diffusion. This was recently addressed by a single-cell encapsulation scheme using alginate microgels designed by [79] in which MSCs were encapsulated in a thin layer of alginate material using a microfluidic approach to form microgels. The reduced size of the encapsulated material allowed for improved diffusive drug transport. This method can enable intravenous drug delivery. The half-life of encapsulated cells is also considerably longer than that of naked cells.

As early as 2016, studies have shown that hydrogel-encapsulated stem cells can be used to treat AD mice. Other materials like DSP (a self-assembled peptide) can promote neuroprotection and exert anti-neuroinflammation and paracrine effects by increasing the survival rate and differentiation ratio of transplanted stem cells, thereby maximizing the therapeutic effect of NSC transplantation for AD [26].

The potential for widespread application or commercialization of such technology is enhanced by the fact that encapsulants can be preserved using normal cryopreservation techniques and defrosted cells have similar persistence of to freshly encapsulated cells after removing the encapsulants. [155] established a novel hydrogen-bonded (HOFs) encapsulation technique for NSCs in organic frameworks. They concluded that the main causes of the suboptimal efficiency of transplantation are “stemness” loss, cell membrane damage during transplantation, and apoptosis induced by oxidative stress after transplantation. This study was the first to encapsulate NSCs using HOFs and showed that this method can improve NSC viability, encourage neurogenesis, and reduce cognitive damage. Protective encapsulation of stem cells may be a viable means to prevent loss during transplantation and guarantee efficiency. [154] established a delivery strategy for encapsulated NSC drugs that integrates antioxidant nanoenzymes (e.g., ceria) into metal–organic frameworks (MOFs). This method combines the antioxidant capacity of nanoenzymes with the effective drug delivery of MOFs, resulting in lower oxidative damage to stem cells while the reactive framework releases encapsulated stem cells in the lesion area. Targeted delivery with a reactive framework while loading enzymes to prevent oxidative damage is instructive as a delivery route.

MOFs have also recently attracted attention due to their potential in developing new AD diagnostic methods. MOFs are assessed by fluorescence and electrochemiluminescence (ECL) detection of AD biomarkers. Fluorescence detection of major metal ions in the brain (Zn^2+^, Cu^2+^, Fe^3+^, and Al^3+^) can be performed as part of magnetic resonance imaging (MRI) [124].

In summary, encapsulation methods and their modifications appear to be viable and adaptable strategies to achieve targeted release of stem cells and increase the survival rate. The reactive design of the encapsulating framework enables targeted release of stem cells, while the varying loads (enzymes, etc.) provide the potential to withstand oxidative damage. What is currently needed is to adjust the size of the formed package carriers (e.g., to form microgels) with reference to existing studies to create an encapsulating vehicle that is appropriate for intravenous drug delivery while avoiding issues like vascular blockage. The optimum method of encapsulation should enable post-thaw use in addition to conventional cryopreservation.

**Regulation of gene expression in stem cells**

A commonly used method to alter cellular phenotypes is the introduction of genes encoding specific products. At present, many studies use viruses as vectors for this process. For stem cell therapy for neurological diseases, a promising option is to upregulate gene expression to help localize post-transplanted stem cells to the lesion sire. [152] demonstrated in vitro that overexpression of *Wnt3a* promoted the migration and neural differentiation of MSCs through the Wnt/PCP pathway. Although in vivo experiments are needed to demonstrate whether upregulating expression of this pathway has the same effect, this method has the potential to promote MSC migration to achieve better efficacy. [152]

##### Investigation of alternative, ethically-acceptable sources of stem cells

At present, the main sources of stem cells can be grouped into four categories: embryonic tissue, fetal tissue, specific locations in adult organisms, and differentiated somatic cells after being genetically reprogrammed [7]. A significant proportion of these sources involve the destruction of embryos or fetuses, which has historically been a source of ethical controversy in stem cell therapy. Of these, only morula-derived ESCs are totipotent, i.e., can differentiate into all cell types in an organism. In contrast, stem cells from fetuses are generally pluripotent and can differentiate into a limited number of specific cell types. To circumvent the ethical issues of extracting stem cells, iPSCs seem to be a promising source of stem cells. However, there is the issue of overcoming possible immune rejection caused by allogeneic transplantation. Using a patient’s own somatic cells as a source of stem cells would result in an increased time cost and is not suitable for large-scale clinical application. Currently, adipose-derived stem cells (ASCs) are one of the best choices for stem cell therapy and tissue engineering due to their easy accessibility and proliferative capacity relative to BM-MSCs. In the treatment of neurodegenerative diseases, ASCs have been observed to outperform various other stem cells in the secretion of trophic factors such as BDNF. Therefore, adipose tissue appears to be a good source of stem cells for stem cell-based therapy considering ease of access, ethical issues, and overall efficacy.

Overcoming the current limitations of stem cell technology is crucial for the advancement of AD research and treatment. The improvement and implementation of new strategies will enhance the quality and utility of stem cell-based platforms for AD research and therapy, leading to better outcomes for patients with AD and related dementias.

### Limitations and strategies of stem cell-derived exosome therapy

Despite the great potential of exosome-based therapies, many difficulties must still be overcome to translate these therapies into clinical practice. First, traditional EV production processes based on pricey 2D cell culture protocols require a lot of cell culture plastic, media, and space. Furthermore, as exosomes are secretory products of cells, their yield is low and they are difficult to produce on an industrial scale. Another significant obstacle is how to accurately identify exosomes from other extracellular vesicles (EVs), particularly functional microvesicles. The techniques that aim to address these challenges are described in the following section [29].

#### Increasing exosome secretion

##### 3D culture

Traditional 2D culture techniques are less efficient in terms of space, material, and yield. In contrast, 3D culture offers healthier culture conditions and has the potential to produce higher EV secretion rates. MSC-EVs from conventional 2D monolayer cell cultures, 3D suspended droplet spheroids, and 3D aggregates were compared by Harrel et al. [48]. Their results showed that EV secretion was three-fold higher using 3D culture compared to 2D culture. Thus, an effective way to improve exosome production is by 3D culture.

A recent study that treated AD mice with 3D-MSC-EVs found that when 6E10 antibodies were used as the criterion to assess Aβ density, there was no significant difference in intracellular levels of HPCs between the control group and 3D-MSC-EV group; however, the levels of some inflammatory markers in mice’s brains were significantly reduced and behavioral experiments yielded positive results [24].

##### Chemical molecular interference

Inducing metabolic changes and other disruptions in cells through the administration of chemical molecules can also impact exosome generation. By blocking both oxidative phosphorylation and glycolysis, sodium iodoacetate (IAA) and 2,4-dinitrophenol (DNP) were shown to effectively reduce the cellular energy charge. Exosome production can also be increased using chemicals like chloroquine, lipocalin (APN), and NH4Cl [24].

##### Environmental factors

According to previous studies [54, 148], exosomes can be promoted by hypoxia, pH changes, and low glucose levels. EV secretion was slightly enhanced after moderate hypoxic treatment of MSCs (0.5–1% oxygen), while no increase in exosome release was observed when MSCs were stimulated with 5% hypoxia, although the nature of the exosomes was altered. Many researchers are interested in acidity as a potential way to control exosome generation. A recent study demonstrated that the environmental factors of excessive acid and alkali resulted in decreased exosome formation compared to normal pH levels [24].

Studies have also shown that MSCs in a hypoxic state present stronger activity, while oxygen-rich environments lead to lower stage viability of MSCs. [69] administered hypoxic-conditioned MSC-derived miR-21-riched exosomes to APP/PS1 mice. Their results showed that hypoxia-treated MSCs had obvious activity, while upregulation of miR-21 enhanced memory and reduced Aβ plaque deposition. [69]

#### Improvement in separation methods

Exosomes, which are a part of stem cell secretomes that differs from microvesicles and apoptotic vesicles, occur through direct germination of the plasma membrane, whose biogenesis starts with inward growth of the plasma membrane to create early endosomes. These endosomes then mature into multivesicular bodies (MVBs), including inward germination through the endosomal membrane, to create intraluminal vesicles (ILVs).

The emerging role of exosomes in AD research and therapy has created the need for their large-scale isolation. However, this is costly and difficult using conventional methods like ultra-centrifugation. Ultrafiltration-based exosome separation is a good substitute for conventional ultracentrifugation because it involves a shorter processing time and requires no specialized equipment. Additionally, by varying the size of the filter, ultrafiltration enables separation and collection of tiny extracellular vesicles of a certain particle size, including exosomes. Tangential flow filtration is another efficient method to eliminate vesicle blockage caused by ultrafiltration in combination with pretreatment with protease, resulting in decreased viscosity of the fluid sample. Microfluidic devices are ideal tools for exosome separation and are beginning to achieve acceptance as superior exosome detection tools. These compact platforms make it possible to process nanovesicles quickly and affordably—even in small quantities of liquid samples.

A different technique for separating exosomes while maintaining the biological activity of isolated exosomes is volumetric exclusion (SEC). Unlike ultra-ionization and ultrafiltration, SEC is achieved by passive gravity flow with no effects on vesicle structure and integrity. High resolution and repeatability are additional benefits of SEC [150, 219].

## Conclusions and future directions

This review provides a comprehensive overview of the latest research advancements in stem cell-derived models for AD and discusses the fundamental mechanisms and applications of stem cells and their derived therapies for mitigating AD pathology. Stem cell technology is helpful for the discovery of such mechanisms and potential therapeutic options, including cell transplantation and generating exosomes for disease treatment.

Our understanding of the pathogenic mechanisms of AD has improved with ongoing research, progressing from the original amyloid theory to higher levels including immunological and genetic theories. Increased knowledge of pathogenic mechanisms has helped researchers identify new drug targets, and the rapid development of stem cell technology has made it possible to reveal underlying mechanisms of AD pathology previously unavailable. For example, iPSC-derived disease models, as well as humanized AD animal models, have great potential for drug development and AD screening. Due to the limitations of 2D and 3D stem cell model protocols, there are still no models that can fully simulate the brains of fAD patients. Thus, in further research, aging and environmental factors should be included in iPSC-based models to explore whether phenotypes occur only in patient-specific cells.

To generate cell–cell circuits of different cell types, co-culture systems, microfluidic chips, and 3D organoids containing neurons, glial cells, and immune cells with complex neuronal networks mimicking the brain should be developed. Researchers need to clarify the key principles of delaying aging to reverse AD through epigenetic remodeling. Combined with multi-dimensional phenotypic analysis and single-cell transcriptome sequencing, the interactive effects of tissue injury, inflammation, and regeneration on the structure of the nervous system and systemic vasculature, aging, and immune challenge need to be systematically studied. A systematic view of AD needs to be achieved through complex data modeling. AD organoid models constructed using gene editing tools to assess the association between risk genes and disease phenotypes require further research. Although CRISPR has recently been useful in gene treatment of AD, its applications in humans are mainly limited to cancer research.

Notably, most existing attempts to treat AD with stem cells have used a single type of stem cell. Given the complexity of human neural networks, stem cell transplantation may need to be performed in conjunction with small molecule drug induction. For example, in the case of neurogenesis, there is a reciprocal regulation between drug-induced regeneration and stem cell-induced regeneration, and the state of supporting cells in the environment and the use of stem cells to induce glial cells for transplantation therapy also show considerable potential. Combined application of stem cells and glial cells is also a promising direction. In the future, it seems likely that stem cell therapies will be combined with small molecule drugs (that promote neuroregeneration or regulate differentiation) or glial cell precursors. Such combination therapies can be studied accurately and efficiently using the above-described multi-level and multi-faceted models.

Many recently developed omics technologies already have clinical applications. AD is a complex disease, and its pathogenesis is caused by protein alterations as well as DNA, mRNA, and even miRNA alterations. Research on the pathogenesis and treatment of AD is thus increasingly adopting a multi-omics approach. Take stem cell-derived exosomes as an example. The exosomes contain proteins, mRNAs, miRNAs, and other substances, and their contents can be changed in a targeted manner through exosome engineering, resulting in a therapeutic effect on AD from a multi-omics perspective. Researchers have identified many proteins that contribute to neuroprotection in MSC-derived exosomes, and many miRNAs that can be encapsulated in exosomes—such as miR-29a, miR-126-3p, miR-137, miR-144-3p, miR-188-5p, miR-425-3p, and miR-451a—can have therapeutic effects on AD. Such findings represent new advancement in multi-omics research in the exploration of AD mechanisms and therapeutic approaches.

The majority of drugs and therapies for AD focus on the late stage of AD onset, which is characterized by memory loss and aphasia that seriously impact the lives of AD patients and their families. Medication for advanced AD mainly aims to alleviate symptoms, reduce clinical progression, and delay disability. However, drugs with the potential to prevent AD are being tested using stem cell-derived disease models. Stem cell models make it possible to demonstrate the entire disease process, reflecting effects of environmental factors and indicators of pathogenic risks. Regular physical examinations before clinical symptoms appear and behavioral diagnosis may indicate AD development. Stem cells and their derivative therapies may also be able to intervene at the early stages of AD to provide a real cure rather than just alleviate symptoms and delay progression, providing a new way of thinking about treating AD and reducing the burden of this disease on the elderly and society.

The life sciences have entered the era of big data, big platforms, and big discoveries. Big data technology has greatly enhanced the efficiency of research and biotechnology for the treatment of AD. Modern software, machine learning, and comprehensive data analysis methods can aid in the analysis of complex biological processes involved in aging and disease and help evaluate the efficacy and safety of pharmacological interventions and cell or cell-derived exosome therapies. Thus, interdisciplinary collaborations between researchers from different fields are crucial.

Personalized medicine and precision medicine of biomarker development have also radically changed the conventional paradigm of disease diagnosis and treatment, resulting in transformation of the pharmaceutical industry in the field of AD. Stem cells and regenerative medicine have opened up new paths for disease treatment. Novel research methods, such as single-cell technology, directed proteomics technology, genome editing technology, and optogenetics technology, have enabled exploration of AD in a more precise and real-time manner.

In conclusion, stem cell technology has the potential to significantly advance the understanding and treatment of AD by providing new disease models, drug development opportunities, and cell therapy options. In the future, stem cell technology will develop in the direction of informatization, manipulation, and multi-omics and play a positive role at different levels to achieve better therapeutic results for AD.

## Data Availability

Data sharing not applicable to this article as no datasets were generated or analysed during the current study.
